# Two PI 3-Kinases and One PI 3-Phosphatase Together Establish the Cyclic Waves of Phagosomal PtdIns(3)P Critical for the Degradation of Apoptotic Cells

**DOI:** 10.1371/journal.pbio.1001245

**Published:** 2012-01-17

**Authors:** Nan Lu, Qian Shen, Timothy R. Mahoney, Lukas J. Neukomm, Ying Wang, Zheng Zhou

**Affiliations:** 1 Verna and Marrs McLean Department of Biochemistry and Molecular Biology, Baylor College of Medicine, Houston, Texas, United States of America; 2 Department of Molecular and Human Genetics, Baylor College of Medicine, Houston, Texas, United States of America; 3 Institute of Molecular Life Science, University of Zürich, Zürich, Switzerland; 4 Program in Developmental Biology, Baylor College of Medicine, Houston, Texas, United States of America; Dartmouth Medical School, United States of America

## Abstract

Phosphatidylinositol 3-phosphate (PtdIns(3)P) is a signaling molecule important for many membrane trafficking events, including phagosome maturation. The level of PtdIns(3)P on phagosomes oscillates in two waves during phagosome maturation. However, the physiological significance of such oscillation remains unknown. Currently, the Class III PI 3-kinase (PI3K) Vps34 is regarded as the only kinase that produces PtdIns(3)P in phagosomal membranes. We report here that, in the nematode *C. elegans*, the Class II PI3K PIKI-1 plays a novel and crucial role in producing phagosomal PtdIns(3)P. PIKI-1 is recruited to extending pseudopods and nascent phagosomes prior to the appearance of PtdIns(3)P in a manner dependent on the large GTPase dynamin (DYN-1). PIKI-1 and VPS-34 act in sequence to provide overlapping pools of PtdIns(3)P on phagosomes. Inactivating both *piki-1* and *vps-34* completely abolishes the production of phagosomal PtdIns(3)P and disables phagosomes from recruiting multiple essential maturation factors, resulting in a complete arrest of apoptotic-cell degradation. We have further identified MTM-1, a PI 3-phosphatase that antagonizes the activities of PIKI-1 and VPS-34 by down-regulating PtdIns(3)P on phagosomes. Remarkably, persistent appearance of phagosomal PtdIns(3)P, as a result of inactivating *mtm-1*, blocks phagosome maturation. Our findings demonstrate that the proper oscillation pattern of PtdIns(3)P on phagosomes, programmed by the coordinated activities of two PI3Ks and one PI 3-phosphatase, is critical for phagosome maturation. They further shed light on how the temporally controlled reversible phosphorylation of phosphoinositides regulates the progression of multi-step cellular events.

## Abstract 2

Cyclic oscillations in the level of phosphatidylinositol 3-phosphate in phagosomes, regulated by two phosphoinositide kinases and one phosphatase, are critical for phagosome maturation and degradation of apoptotic cells.

## Author Summary

During animal development and in adulthood many cells are programmed to die by an active process called apoptosis. These dead or dying apoptotic cells are swiftly taken up by scavenger cells into membrane-bound compartments—phagosomes—where they are subsequently degraded when other intracellular organelles containing digestive enzymes fuse with phagosomes—a process called phagosome maturation. Phagocytosis of apoptotic cells is important for tissue remodeling in development and to prevent harmful inflammatory and autoimmune responses. In nematode worms—a model organism in which to study apoptosis—phagosome maturation is accompanied by two waves of the signaling molecule phosphatidylinositol 3-phosphate (PtdIns(3)P) in this compartment: one that forms soon after the formation of the phagosome and lasts for 10–15 minutes, and a second, weaker one 10 minutes later that lasts until the apoptotic cell is fully digested. In this study, we investigated the mechanism that regulates the timing and length of these two waves. We found that they are established by the sequential and combined action of three enzymes: two phosphoinositide 3-kinases, which add a phosphate group to the 3′ site of PtdIns, and one phosphoinositide 3-phosphatase, which removes it. We showed that inactivation of both kinases depleted phagosomes of PtdIns(3)P and resulted in the arrest of phagosome maturation and degradation of apoptotic cells. In addition, the timely turnover of PtdIns(3)P catalyzed by the phosphatase was critical for the step-wise progress of phagosome maturation. Our findings suggest that reversible phosphorylation of phophoinositides, catalyzed by distinct sets of kinases and phosphatases, might be a general mechanism to drive multi-step intracellular membrane trafficking events.

## Introduction

PtdIns(3)P is a phosphorylated phosphatidylinositol (PtdIns) species that is embedded in distinct membrane domains and plays important roles in many membrane trafficking events, including endocytic trafficking, retrograde trafficking, autophagy, and phagosome maturation (reviewed in [1],[2]). PtdIns(3)P activates downstream pathways through recruiting specific PtdIns(3)P-binding proteins, the PtdIns(3)P effectors, to the site of action [3]. The production and elimination of PtdIns(3)P on a particular membrane domain, such as the surface of phagosomes, are presumably under tight temporal regulation to allow the occurrence of multiple transient signaling events. However, the molecular mechanisms behind such regulation are not well understood.

In metazoan, such as the nematode *Caenorhabditis elegans*, the fruit fly *Drosophila melanogaster*, and mammals, a large number of cells undergo apoptosis during development and adulthood and are subsequently engulfed by phagocytes and degraded inside phagosomes. Like other phagosomal cargos, dying cells are degraded through phagosome maturation, a process that relies heavily on the fusion between phagosomes and various intracellular organelles including endosomes and lysosomes, which leads to the delivery of digestive enzymes into phagosomes and the acidification of the phagosomal lumen [4]–[6]. Immediately after an apoptotic cell is internalized, a high level of PtdIns(3)P appears transiently on the surface of a nascent phagosome [7],[8]. This prominent feature was observed on nascent phagosomes containing various kinds of cargos, including zymosan particles, latex beads, and invading pathogens in addition to apoptotic cells and is well conserved in different organisms [7]–[13]. In *C. elegans* embryos, during the maturation of phagosomes containing apoptotic cells, PtdIns(3)P is dynamically enriched on phagosomes in two consecutive waves: the initial burst, which appears upon the closure of a phagocytic cup and dissipates after 10–15 min, and a subsequent reappearance of a relatively weaker PtdIns(3)P signal approximately 10 min later, which lasts until an apoptotic cell is fully degraded [7],[8]. Such a PtdIns(3)P oscillation pattern has also been observed on phagosomes containing other kinds of cargos [10],[14]. However, the physiological significance of this biphasic PtdIns(3)P appearance on phagosomes remains unknown.

PtdIns(3)P is a 3′-phosphorylated form of PtdIns. Among the three classes of known phosphoinositide 3-kinases (PI3Ks), Vps34, the sole Class III PI3K, is known to specifically produce PtdIns(3)P on intracellular membranes, such as endosomes, phagosomes, and autophagosomes, to regulate diverse membrane trafficking events (reviewed in [2],[15]). Specific inactivation of Vps34, through injection of anti-Vps34 antibodies into mammalian cells or RNA interference (RNAi) treatment in *C. elegans*, reduces the efficiency of phagosome maturation, indicating that PtdIns(3)P is important for promoting phagosome maturation [12],[13],[16]. However, whether Vps34 is the only PI3K that produces PtdIns(3)P on phagosomal surfaces remains unknown. Furthermore, in the absence of a strategy capable of complete depletion of phagosomal PtdIns(3)P, it is impossible to quantitatively determine how fundamental the role of PtdIns(3)P is in initiating phagosome maturation.

During the development of *C. elegans* hermaphrodites, 131 somatic cells and approximately 300–500 germ cells undergo programmed cell death [17]. These cells are easily recognizable within living animals under the Nomarski Differential Interference Contrast (DIC) optics as highly refractive, button-like objects referred to as “cell corpses” [18],[19]. In *C. elegans*, after being rapidly engulfed and contained inside phagosomes, apoptotic cells are degraded via a pathway initiated by the phagocytic receptor CED-1, which is transiently clustered on the surface of extending pseudopods and nascent phagosomes [8]. CED-1 triggers the robust production of PtdIns(3)P on phagosomal surfaces through recruiting the large GTPase DYN-1, the *C. elegans* homolog of mammalian dynamins, to phagosomes [8],[20]. In another study focusing on how PtdIns(3)P triggers phagosome maturation in *C. elegans*, we identified LST-4/SNX-9, SNX-1, and SNX-6, three PX and BAR domain-containing sorting nexins, as PtdIns(3)P effectors recruited to phagosomal surfaces by PtdIns(3)P and subsequently acting in two parallel pathways to drive the incorporation of endosomes and lysosomes into phagosomes [21]. These findings revealed a signaling cascade initiated by phagocytic receptor CED-1, mediated by PtdIns(3)P, and executed through these sorting nexins to degrade apoptotic cells [8],[21].

Interestingly, the phagosome maturation-delay/arrest phenotype observed from *C. elegans vps-34* mutants was much milder than that from the *ced-1* or *dyn-1* single mutants, or the *lst-4*; *snx-1*; *snx-6* triple mutants [8],[13],[21], suggesting that inactivating VPS-34 alone might not completely deplete PtdIns(3)P from phagosomes. We examined whether there existed additional PI 3-kinase(s) responsible for producing phagosomal PtdIns(3)P.

Besides Vps34, Class II PI3Ks are also able to produce PtdIns(3)P from PtdIns, whereas Class I PI3Ks primarily produce PtdIns(3,4,5)P_3_ [15]. In vitro, PtdIns is the favorable substrate for Class II PI3Ks [22]–[25]. Multiple lines of evidence have revealed that Class II PI3Ks produce PtdIns(3)P in vivo in response to certain extracellular and intracellular stimuli [26]–[29]. However, in comparison to Class I and III PI3Ks, relatively little is known about the physiological functions and regulation of Class II PI3Ks (reviewed in [15],[30]). In particular, it is not known whether any Class II PI3K(s) is involved in producing PtdIns(3)P on phagosomes. Here we revealed the function of PIKI-1, the only *C. elegans* Class II PI3K, in phagosome maturation.

In a closely related aspect, little is known about the factors that down-regulate the initial peak of phagosomal PtdIns(3)P, or the physiological significance of PtdIns(3)P oscillation during phagosome maturation. Promising candidates able to down-regulate PtdIns(3)P level include PtdIns(3)P phosphatases. The myotubularin phosphatases are a family of PI phosphatases that convert PtdIns(3)P or PtdIns(3,5)P_2_ into PtdIns or PtdIns(5)P, respectively [31]. They act to maintain the homeostasis of PtdIns(3)P on intracellular membranes [29],[31]. Inactivation of MTM-1, a member of the myotubularin family in *C. elegans*, rescued the endocytosis defect of *vps-34* mutants, suggesting that MTM-1 antagonizes the PI 3-kinase activity of VPS-34 during endosomal trafficking [32]. However, it was not known whether MTM-1 was involved in the dephosphorylation of phagosomal PtdIns(3)P.

In this report, we identified a novel role of the *C. elegans* Class II PI3K PIKI-1 in the degradation of apoptotic cells. We further revealed the differential and complementary roles of PIKI-1 and VPS-34 in the production and maintenance of phagosomal PtdIns(3)P. Moreover, we have uncovered a novel function of MTM-1 in modulating the dynamic pattern of PtdIns(3)P on phagosomes. We found that the prompt down-regulation of phagosomal PtdIns(3)P, like its robust production on nascent phagosomes, is pivotal for driving phagosome maturation. Our work revealed a regulatory system that issues a precise temporal control over the PtdIns(3)P cycling pattern on phagosomes and ensures the efficient degradation of apoptotic cells.

## Results

### Inactivation of Class III PI3K VPS-34 Only Partially Depletes PtdIns(3)P from Phagosomal Surfaces

To evaluate whether inactivating *vps-34* would result in a complete depletion of phagosomal PtdIns(3)P, we monitored the level of PtdIns(3)P on the surface of phagosomes using a previously established PtdIns(3)P reporter, 2xFYVE::GFP, which was expressed in engulfing cells under the control of P*_ced-1_* and specifically associated with PtdIns(3)P [8],[33]. We first monitored phagosomal PtdIns(3)P inside gonadal sheath cells, the engulfing cells for apoptotic germ cells [34].

**Figure 1:**
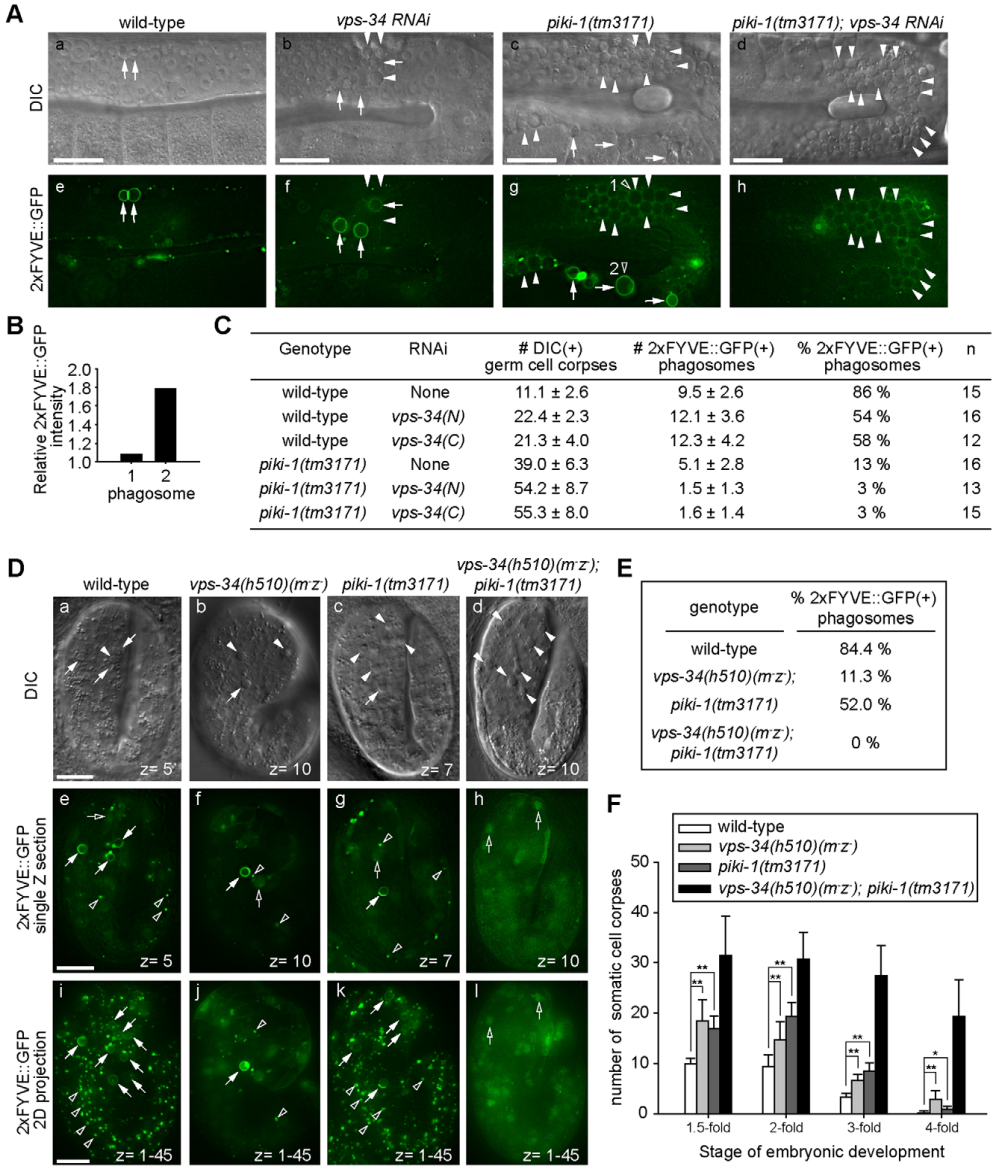
VPS-34 and PIKI-1 act together to produce a robust amount of PtdIns(3)P on phagosomes and drive the degradation of apoptotic cells. (A–C) Wild-type and mutant adult hermaphrodites analyzed here all carried P*_ced-1_*2xFYVE*::gfp* expressed in gonadal sheath cells and were analyzed 48 h after the mid-L4 stage. RNAi treatments were started at L4 stage. (A) DIC (a–d) and epifluorescence images (e–h) of gonad arms of wild-type or mutant adult hermaphrodites. Dorsal is to the top. Arrows and arrowheads indicate 2xFYVE::GFP(+) and 2xFYVE::GFP(−) phagosomes, respectively. Open arrowheads in (g) indicate one 2xFYVE::GFP(−) and one 2xFYVE::GFP(+) phagosome, whose relative GFP signal intensities were shown in (B). Scale bars, 20 µm. (B) The ratio of GFP intensity on phagosomal surface/GFP intensity in the cytosol of the engulfing cell of the two phagosomes marked with open arrowheads in A(g). (C) The numbers of germ cell corpses per gonadal arm and percentage of 2xFYVE(+) phagosomes. *vps-34*(N) and *vps-34*(C) are two independent RNAi feeding constructs targeting different regions of *vps-34* coding sequence. Data are presented as mean ± standard deviation (SD). *n*, number of animals scored. (D–F) “*vps-34*(*m* ^−^ *z* ^−^)” indicates the genotype of embryos in which both the maternal and zygotic products of *vps-34* were inactivated. (D) DIC (a–d) and epifluorescent images (e–l) of 2-fold stage embryos expressing P*_ced-1_*2xFYVE*::gfp*. Genotypes are indicated on top of the images. (e–h) Single z-sections (section number labeled) of GFP images that correspond to the DIC images in (a–d). (i–l) 2-D projections of 45 serial *z*-sections (at 0.5 µm intervals) of GFP images that span the entire depth of the embryos shown in (a–h). Filled arrows and arrowheads indicate 2xFYVE::GFP(+) and 2xFYVE::GFP(−) cell corpses, respectively. Open arrowheads indicate PtdIns(3)P(+) endosomes. Open arrows indicate nuclei that were labeled by 2xFYVE::GFP. Scale bars, 10 µm. (E) The Percentage of 2xFYVE::GFP(+) phagosomes in 2-fold stage embryos carrying P*_ced-1_*2xFYVE*::gfp*. At least 200 phagosomes were scored for each genotype. (F) The numbers of somatic cell corpses in embryos at different stages with indicated genotypes. These embryos did not carry P*_ced-1_*2xFYVE*::gfp*. At least 20 animals were scored for each data point. Data are presented as mean ± SD. * *p*<0.05 and **p<0.001 calculated by independent Student's *t*-test.

In the gonad of wild-type adult hermaphrodites, 86% of germ cell corpses, recognized under the DIC optics, were labeled with bright 2xFYVE::GFP on their surfaces (Figure 1A(a, e) and C), indicating the presence of PtdIns(3)P at high levels on phagosomal surfaces. Knocking down *vps-34* by RNA interference (RNAi) using two non-overlapping RNAi constructs reduced the percentage of gonadal phagosomes labeled with 2xFYVE::GFP to 54% or 58%, respectively (Figure 1A(b, f) and C). Furthermore, *vps-34*(RNAi) animals displayed a mild cell-corpse removal defective (Ced) phenotype resulting from un-removed cell corpses, evident by the modestly increased numbers of germ cell corpses in the gonad (Figures 1C and S1A), as reported previously [13]. These results suggest that inactivation of *vps-34* only partially impairs the production of PtdIns(3)P on gonadal phagosomes.

To examine VPS-34's contribution to the production of phagosomal PtdIns(3)P in a more strict manner, we created a strain that produced *vps-34(h510)*(*m* ^−^ *z* ^−^) (m, maternal gene product; z, zygotic gene product) homozygous null mutant embryos that lost both the maternal and the zygotic expression of *vps-34*, which is likely to represent the *vps-34* complete loss-of-function phenotypes (Figure S1B and Text S1).

In *vps-34(h510)*(*m* ^−^ *z* ^−^) mutant embryos, the number of PtdIns(3)P-labeled cytoplasmic puncta, which represented early endosomal particles, was nearly abolished, consistent with the known function of VPS-34 in the production of PtdIns(3)P on early endosomes (Figure 1D(e, f, i, j)) [35]. Moreover, PtdIns(3)P was much less frequently detected on phagosomal surfaces than in wild-type embryos (Figure 1D(b, f, j) and E). Consistent with this defect, mutant embryos displayed a modest Ced phenotype, retaining more cell corpses than wild-type embryos at all four embryonic stages examined (Figure 1F). However, as in the gonad, PtdIns(3)P was still detectable on certain phagosomes (11.3%) in embryos (Figure 1D(f) and E), indicating that the inactivation of *vps-34* reduces but does not completely abolish the production of PtdIns(3)P on phagosomes. Additional PtdIns(3)P-producing activity besides VPS-34 must exist on phagosomal membranes.

### Identification of a Novel PI3K That Produces PtdIns(3)P on Phagosomes

**Figure 2:**
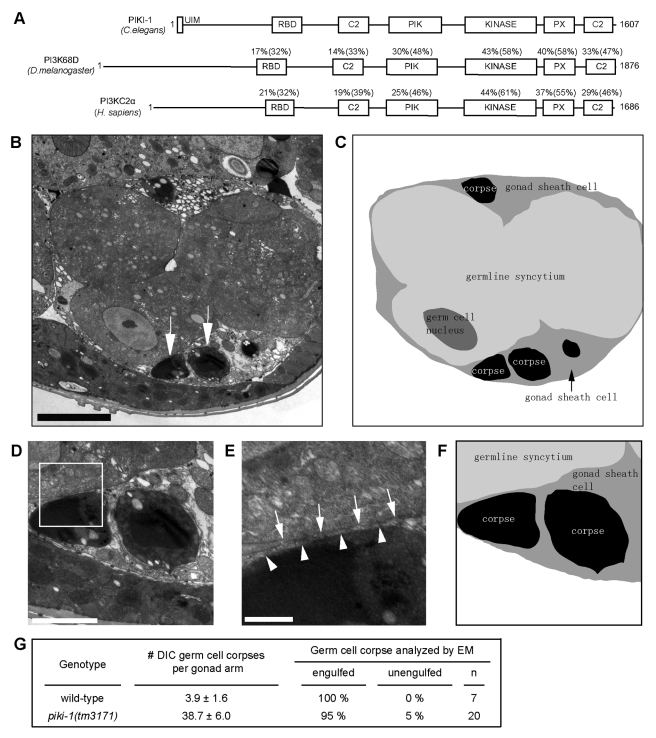
In the gonad of *piki-1(tm3171)* mutants, apoptotic germ cells are engulfed but fail to be degraded. (A) Domain structure of PIKI-1 and its orthologs in other species. Percentage indicates amino acid identity (similarity in parentheses) of each domain between PIKI-1 and its orthologs; UIM, ubiquitin-interacting motif; RBD, Ras-binding domain; C2, protein kinase C conserved region 2; PIK, phosphoinositide 3-kinase, accessory domain; Kinase, phosphoinositide 3-kinase, catalytic domain; PX, PhoX homologous domain. (B–C) A cross-section transmission electron microscopy (TEM) image of a distal gonad arm in a *piki-1(tm3171)* adult hermaphrodite (B) and its corresponding traces of membranes (C). Cell identities are labeled. White arrows in (B) indicate two engulfed cell corpses. The black arrow in (C) indicates a gonadal sheath cell. Scale bar: 5 µm. (D–F) Enlarged images of germ cell corpses indicated by arrows in (B) and their surrounding environment inside a sheath cell are displayed in (E). (F) Schematic diagram of (D). (E) Further enlarged image of the framed region in (D). White arrows and arrowheads mark the plasma membranes of gonadal sheath cells and germ cell corpses, respectively. Scale bars: 2 µm in (D); 0.5 µm in (E). (G) Percentage of engulfed and unengulfed germ cell corpses quantified by TEM. Data of wild-type animal are from [20]. *n*, number of germ cell corpses analyzed.

Previously, we found that *tm3171*, a deletion mutation of *piki-1*, which encodes the only *C. elegans* Class II PI3K (Figure 2A, Figure S2A, Text S1), resulted in a strong Ced phenotype in the adult hermaphrodite gonad (Figure 1A(c) and C) [21]. We further found that the *piki-1(tm3171)* mutation also resulted in moderate Ced phenotypes in embryos throughout mid- and late-embryonic stages (Figure 1F). These results indicate that *piki-1* is required for the removal of both the somatic and germ cell corpses.

In the gonad of *piki-1(tm3171)* mutant hermaphrodites, the percentage of phagosomes labeled with 2xFYVE::GFP was reduced to 15% of the wild-type level (Figure 1A(c, g), B–C). The majority of gonadal phagosomes were embedded inside the 2xFYVE::GFP-expressing cytoplasm of the host cells, yet lacked enriched GFP signal on their surfaces, appearing as “dark spheres” (Figure 1A(g, filled arrowheads) and B). These observations revealed a novel activity of PIKI-1 in producing phagosomal PtdIns(3)P. Moreover, quantitative comparison of the percentage of PtdIns(3)P-labeled phagosomes in *piki-1(tm3171)* and *vps-34*(RNAi) animals indicates that in gonadal sheath cells, PIKI-1 plays a major role in producing PtdIns(3)P on phagosomes (Figure 1C).

In mid-stage (2-fold stage) *piki-1(tm3171)* mutant embryos, the percentage of PtdIns(3)P-labeled phagosomes was reduced to 40% of the wild-type level (Figure 1D and E), indicating that, like in the adult gonad, *piki-1* also plays a role in producing phagosomal PtdIns(3)P in somatic engulfing cells.

Unlike *vps-34(h510)*(m^−^z^−^) mutant animals, which are embryonic and larval lethal (Figure S1B), *piki-1(tm3171)* mutant animals undergo normal development, and are viable and fertile. In addition, unlike *vps-34*(RNAi) animals, *piki-1(tm3171)* animals display normal endocytosis activities (Text S1 and Figure S3) [36]. Consistent with this observation, similar numbers of the bright PtdIns(3)P-labeled puncta, which represent endosomal particles, were found in the cytoplasm in both *piki-1(tm3171)* mutant and wild-type embryos (Figure 1D(i, k)), indicating that the function of PIKI-1 was dispensable for the production of PtdIns(3)P on early endosomes. These results indicate that PIKI-1 specifically produces PtdIns(3)P on phagosomes, and that this activity is required for the efficient removal of cell corpses.

### PIKI-1 Specifically Drives the Degradation But Not the Engulfment of Apoptotic Cells

Inefficient removal of apoptotic cells might be caused by defects in either the internalization step or the degradation step. In *piki-1(tm3171)* mutant adult hermaphrodites, persistent germ cell corpses were all observed inside phagosomes: only 13% of these phagosomes were labeled with PtdIns(3)P on their surfaces that appeared like bright GFP circles, the rest of them lacked PtdIns(3)P on their surfaces and appeared as dark spheres inside the host cells (Figure 1A(c, g) and 1C). This observation suggests that in *piki-1* mutants, cell corpses were successfully engulfed but not efficiently degraded inside phagosomes. Using transmission electron microscopy (TEM), we analyzed the internalization status of germ cell corpses (Materials and Methods) and found that 95% of germ cell corpses in the gonad of *piki-1(tm3171)* mutants were fully engulfed and remained undegraded inside phagosomes (Figure 2B–G). This result confirmed that the function of PIKI-1 is essential for the degradation but not the engulfment of cell corpses.

### Phagosomal PtdIns(3)P Is Indispensible for the Degradation of Apoptotic Cells

Since the inactivation of either VPS-34 or PIKI-1 only partially impaired the PtdIns(3)P production on phagosomes, we further monitored PtdIns(3)P in the gonad of *piki-1(tm3171)*; *vps-34*(RNAi) hermaphrodites and observed a near complete depletion of phagosomal PtdIns(3)P (Figure 1A(d, h) and 1C). Similarly, in mid-stage *vps-34(h510)(m^−^z^−^)*; *piki-1(tm3171)* double mutant embryos, PtdIns(3)P was no longer detected on phagosomal surfaces or on any cytoplasmic puncta (Figure 1D(d, h, l) and E). These observations indicate that VPS-34 and PIKI-1 together produce all the PtdIns(3)P molecules on gonadal and somatic phagosomes. They also indicate that *vps-34*(RNAi) is potent in inactivating VPS-34 in the gonadal sheath cells since only a residual PtdIns(3)P-production activity was left on phagosomes in *piki-1(tm3171)*; *vps-34*(RNAi) double mutant animals.

Previously, without an effective method to completely block the production of PtdIns(3)P on phagosomes, it was impossible to determine whether PtdIns(3)P was absolutely essential for triggering phagosome maturation or merely contributed to phagosome maturation as one of multiple signaling molecules. The *vps-34*; *piki-1* double mutant, in which PtdIns(3)P is no longer detectable on phagosomal surfaces, provides a suitable tool for addressing this question.

In the adult gonads of *piki-1(tm3171)*; *vps-34*(RNAi) double mutants, a larger number of persistent germ cell corpses were observed than in any PI3K single mutants (Figure 1C). As in *piki-1(tm3171)* single mutants, all cell corpses were engulfed inside phagosomes in *piki-1(tm3171)*; *vps-34*(RNAi) double mutants (Figure 1A(d, h, arrowheads), indicating a specific defect in phagosome maturation. Similarly, *vps-34(h510)(m^−^z^−^)*; *piki-1(tm3171)* double mutant embryos displayed a much stronger Ced phenotype than any single mutants (Figure 1F). At 4-fold stage, the number of persistent cell corpses in the double mutant embryos was on average 10-fold larger than that in single mutant embryos (Figure 1F). These observations strongly indicate that PIKI-1 and VPS-34 act in combination to drive the efficient degradation of cell corpses. The tight quantitative correlations between the depletion of phagosomal PtdIns(3)P and the arrest of cell-corpse degradation demonstrate that PtdIns(3)P is an imperative factor for phagosome maturation.

### Phagosomal PtdIns(3)P Is Essential for the Recruitment of SNX-1 and LST-4, Two PtdIns(3)P Effector Proteins, onto Phagosomes

**Figure 3:**
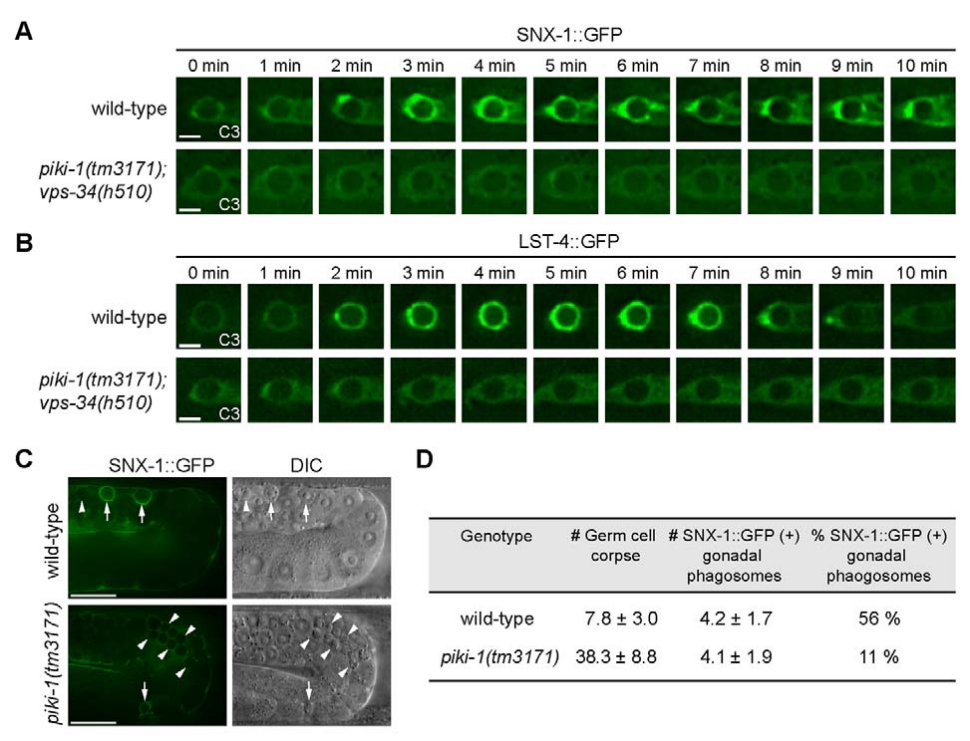
PtdIns(3)P is required for recruiting SNX-1 and LST-4 to the surface of phagosomes. GFP reporters are expressed in the engulfing cells under the control of P*_ced-1_*. (A–B) Time-lapse images showing the dynamic recruitment of SNX-1::GFP (A) or LST-4::GFP (B) onto C3 phagosomes in wild-type embryos or the lack of recruitment in *vps-34(h510)*(*m* ^−^ *z* ^−^); *piki-1(tm3171)* embryos. “0 min” represents the time point when engulfment is just completed. Scale bars, 2 µm. (C) DIC and GFP images of wild-type and *piki-1(tm3171)* mutant adult hermaphrodite gonads expressing SNX-1::GFP in gonadal sheath cells. Arrows and arrowheads indicated phagosomes labeled or not labeled with SNX-1::GFP, respectively. Scale bars, 20 µm. (D) The number of germ cell corpses and percentage of SNX-1::GFP(+) phagosomes in adult hermaphrodites at 48 h after L4 stage. Data are presented as mean ± standard deviation (SD). Fifteen animals were scored for each sample.

Previously, we identified SNX-1, SNX-6, and LST-4/SNX-9, three BAR domain-containing sorting nexins, as novel PtdIns(3)P effectors that drive the degradation of apoptotic cells [21]. In vitro, SNX-1 and LST-4 display high affinity towards PtdIns(3)P [21]. To further determine whether PtdIns(3)P acts as an upstream regulator of SNX-1 and LST-4, we examined the dynamic localization of SNX-1::GFP and LST-4::GFP reporters (Figure S5) [21] on phagosomes in *vps-34(h510)(m^−^z^−^)*; *piki-1(tm3171)* double mutant embryos. We used a previously established time-lapse recording technique to monitor the maturation process of three well-defined phagosomes, C1, C2, and C3, which were formed inside three adjacent hypodermal cells at ∼330 min post-first cleavage (Figure S6C) [8],[33]. This technique enabled us to compare the dynamic localization pattern of a reporter on the exact same phagosome in different genetic backgrounds. In wild-type embryos, as reported [21], SNX-1::GFP and LST-4:GFP were rapidly recruited onto nascent phagosomes immediately after the sealing of phagocytic cups, and remained on phagosomes for ∼10 min (Figure 3A–B). In contrast, in *vps-34(h510)*(*m* ^−^ *z* ^−^); *piki-1(tm3171)* double mutant embryos, SNX-1 and LST-4 failed to accumulate on phagosomes (Figure 3A–B), indicating that the phagosomal PtdIns(3)P is necessary for the recruitment of its effectors to nascent phagosomes. In adult hermaphrodite gonadal sheath cells, we found that the percentage of phagosomes labeled with SNX-1::GFP was reduced to 20% of the wild-type level (Figure 3C–D). This observation indicates that PtdIns(3)P is also responsible for recruiting sorting nexins to phagosomes containing germ cell corpses.

### PIKI-1 Promotes the Recruitment of Three RAB GTPases to Phagosomes

**Figure 4:**
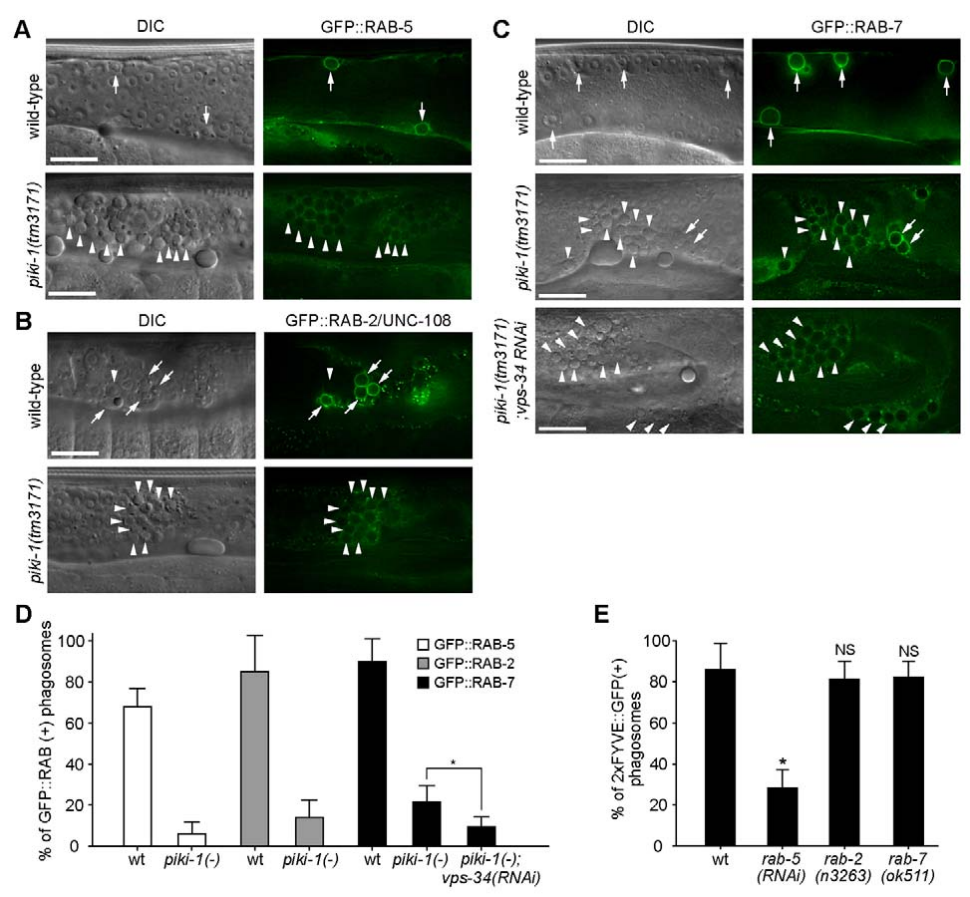
Inactivation of PIKI-1 impairs the recruitment of RAB-5, RAB-2, and RAB-7 to phagosomal surfaces. In addition, RAB-5, but not RAB-2 or RAB-7, is required for the production of PtdIns(3)P on phagosomes. (A–C) DIC and GFP images of gonads of adult hermaphrodite with various genotypes expressing GFP::RAB-5 (A), GFP::UNC-108/RAB-2 (B), or GFP::RAB-7 (C) in the gonadal sheath cells. Animals were analyzed at 48 h after L4 stage. Arrows and arrowheads indicated phagosomes labeled or not labeled with enriched GFP reporter, respectively. Dorsal is to the top. Scale bars, 20 µm. (D) Percentage of phagosomes labeled with various GFP reporters in adult hermaphrodites at 48 h post-L4 stage. Data are presented as mean ± SD. Fifteen animals were scored for each sample. * *p*<0.001 measured by independent Student's *t*-test. (E) The percentage of 2xFYVE::GFP-labeled phagosomes in *rab-5*(RNAi), *rab-2(n3263)*, or *rab-7(ok511)* mutant adult hermaphrodites at 48 h after L4 stages. 15 animals were analyzed for each data point. Data are presented as mean ± SD. * *p*<0.001, independent Student's *t*-test. “NS” indicates non-significant differences (*p*>0.05, independent Student's *t*-test). Images are shown in Figure S4.

To further investigate the function of PtdIns(3)P in regulating phagosome maturation, we examined whether the inactivation of *piki-1* would affect the recruitment of three small GTPases, RAB-5, RAB-2 (also named UNC-108), and RAB-7, to phagosomal surfaces. During phagosome maturation, these membrane-tethering factors are sequentially recruited from the cytoplasm of the host cells to phagosomal surfaces, where they facilitate the fusion of intracellular organelles to phagosomes [7],[8],[13],[37],[38]. In wild-type gonads, we observed that GFP::RAB-5, GFP::RAB-2, and GFP::RAB-7 were enriched on 68%, 85%, and 90% of phagosomes, respectively (Figure 4A–D). In the gonads of *piki-1(tm3171)* mutant hermaphrodites, however, the percentages of phagosomes labeled with GFP::RAB-5, GFP::RAB-2, or GFP::RAB-7 were strongly reduced to 9%, 16%, or 23%, respectively (Figure 4A–D), indicating that the function of PIKI-1 is essential for the recruitment of all three RAB GTPases to phagosomal surfaces. When *piki-1* and *vps-34* were simultaneously inactivated, the phagosomes labeled with GFP::RAB-7 were further reduced to 10% (Figure 4C and D). These results indicate that PtdIns(3)P is essential for recruiting these three RAB proteins.

### RAB-5, but Not RAB-2 or RAB-7, Is Required for the Production of PtdIns(3)P on Phagosomal Surfaces

To further determine the relationships between PtdIns(3)P and the RAB GTPases, we examined the production of PtdIns(3)P on phagosomes in each of the *rab-5*, *rab-2*, and *rab-7* loss-of-function background. Whereas *rab-2* and *rab-7* mutations did not affect the presentation of PtdIns(3)P on phagosomes, inactivation of *rab-5* by RNAi greatly reduced the percentage of PtdIns(3)P-labeled phagosomes (Figures 4E and S4, Text S1), indicating that the production of phagosomal PtdIns(3)P relies on RAB-5 but not RAB-2 or RAB-7. Together, our observations indicate that PtdIns(3)P and RAB-5 depend on each other for the enrichment on the surface of nascent phagosomes. Our observation that PtdIns(3)P and RAB-5 were concurrently enriched on nascent phagosomes further supports this conclusion (Figure S7 and Text S1).

### PIKI-1 and VPS-34 Act Sequentially to Produce PtdIns(3)P on Phagosomes

**Figure 5:**
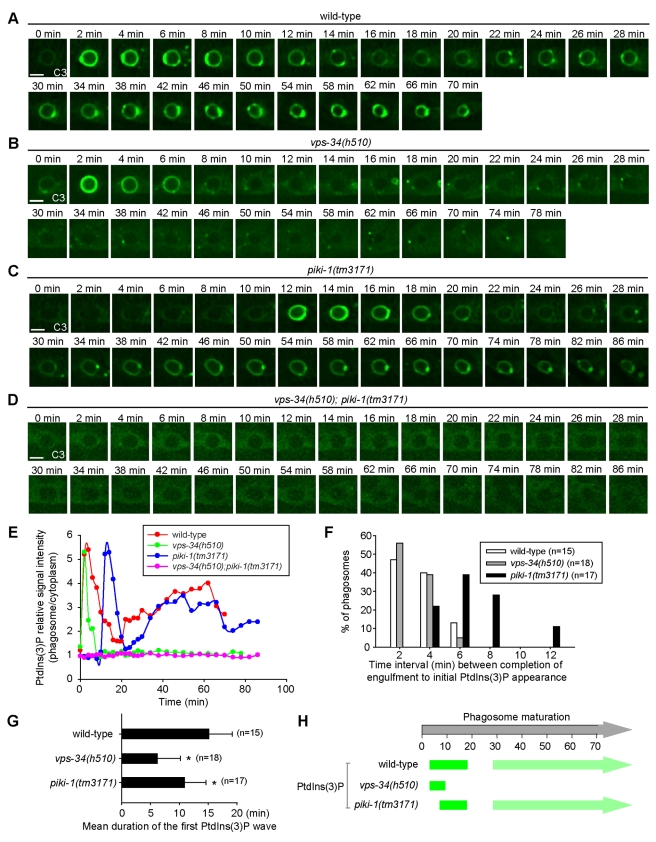
VPS-34 and PIKI-1 produce PtdIns(3)P on phagosomes in differential temporal patterns and, together, are responsible for the total phagosomal PtdIns(3)P production activity. (A–D) Serial time-lapse images displaying the normal or abnormal temporal presentation pattern of PtdIns(3)P on the C3 phagosome during phagosome maturation in wild-type (A), *vps-34(h510)*(*m* ^−^ *z* ^−^) (B), or *piki-1(tm3171)* (C) single mutant embryos, or the lack of phagosomal PtdIns(3)P production in a *piki-1(tm3171); vps-34(h510)*(*m* ^−^ *z* ^−^) double mutant embryo (D). All embryos expressed P*_ced-1_* 2xFYVE*::gfp*. “0 min” represents the time point when C3 was just engulfed. Scale bars, 2 µm. (E) The relative PtdIns(3)P signal intensity, represented as the ratio of PtnIns(3)P signal intensity on the surface of phagosomes to that in the nearby cytoplasm of the host cell, measured from images in (A–D) and plotted over time. (F) Histogram distribution of the time interval between the completion of engulfment and the initial appearance of PtdIns(3)P on phagosomal surfaces, deduced from time-lapse recording of multiple C1, C2, and C3 phagosomes. *n*, number of phagosomes scored. (G) The average duration of the first wave of PtdIns(3)P on phagosomes. Error bars indicate standard deviation. * *p*<0.01, independent Student's *t*-test. *n*, number of phagosomes scored. (H) Diagram summarizing the temporal presentation pattern of PtdIns(3)P on maturing phagosomes in indicated genetic backgrounds. Data represent means obtained from time-lapse recording of multiple C1, C2, and C3 cell corpses, as shown in (E) and (F). The light and dark green colors reflect weak and strong signals, respectively. “0 min” represents the time point when engulfment is just complete.

To determine why the activities of two PI3Ks, which produce an identical signaling molecule PtdIns(3)P on phagosomes, are both needed for efficient phagosome maturation, we monitored the pattern of PtdIns(3)P appearance on phagosomes over time in wild-type, each of the single PI3K mutant, and double PI3K mutant embryos (Figures 5). As reported previously [8], in wild-type embryos, the level of PtdIns(3)P on phagosomes oscillated in a two-wave pattern: immediately (2–4 min) after engulfment, a robust PtdIns(3)P signal rapidly appeared on nascent phagosomes and lasted for ∼15 min before it quickly diminished (Figure 5A,E); after a gap period (∼10 min), a PtdIns(3)P signal weaker than the initial one reappeared on the maturing phagosomes, where it lasted until the phagosomal content was completely degraded (Figure 5A and E). During phagosome maturation, we also frequently observed PtdIns(3)P(+) puncta on phagosomes, which first attached to phagosomal surfaces and later merged into phagosomal membranes (Figure 5A). The dynamic association of PtdIns(3)P puncta with phagosomes likely represents the docking and fusion process of endosomes with phagosomes.

In *vps-34(h510)(m^−^z^−^)*; *piki-1(tm3171)* double mutant embryos, PtdIns(3)P was completely absent from phagosome surfaces throughout the entire recording period (90 min) (Figure 5D and E), causing a complete arrest of phagosome maturation, indicated by the largely unchanged size of phagosomes after a long period of time.

In *vps-34(h510)*(*m* ^−^ *z* ^−^) single mutant embryos, PtdIns(3)P was robustly produced on nascent phagosomes immediately after engulfment, as in the wild-type background (Figure 5B, E, and F). However, PtdIns(3)P disappeared from phagosomal surfaces much more promptly, lasting for only 6 min on average on phagosomes (Figure 5B, E, and G). Moreover, PtdIns(3)P failed to reappear on phagosomes (Figure 5B and E). In contrast, in most *piki-1(tm3171)* single mutant embryos, an obvious delay of PtdIns(3)P production on nascent phagosomes was observed (Figure 5C, E, and F). Furthermore, the duration of the first PtdIns(3)P wave was significantly shorter than that in wild-type control (Figure 5A, C, E, and G). Apart from these aberrations, the disappearance of the initial phagosomal PtdIns(3)P and the subsequent reappearance of PtdIns(3)P on phagosomes followed a relatively normal temporal pattern (Figure 5C and E). These observations indicate that PIKI-1 and VPS-34 play differential roles in the production and maintenance of phagosomal PtdIns(3)P: whereas PIKI-1 is required for the initial production of PtdIns(3)P, VPS-34 is needed for the sustained production of PtdIns(3)P (Figure 5H). Given that the Ced phenotype displayed by *vps-34* and *piki-1* single mutant embryos is similar in severity (Figure 1F), the precise timing of initial PtdIns(3)P production must be as important as the proper time span of PtdIns(3)P appearance on phagosomes for phagosome maturation.

### PIKI-1 Is Recruited to Phagosomal Surfaces in a DYN-1-Dependent Manner

**Figure 6:**
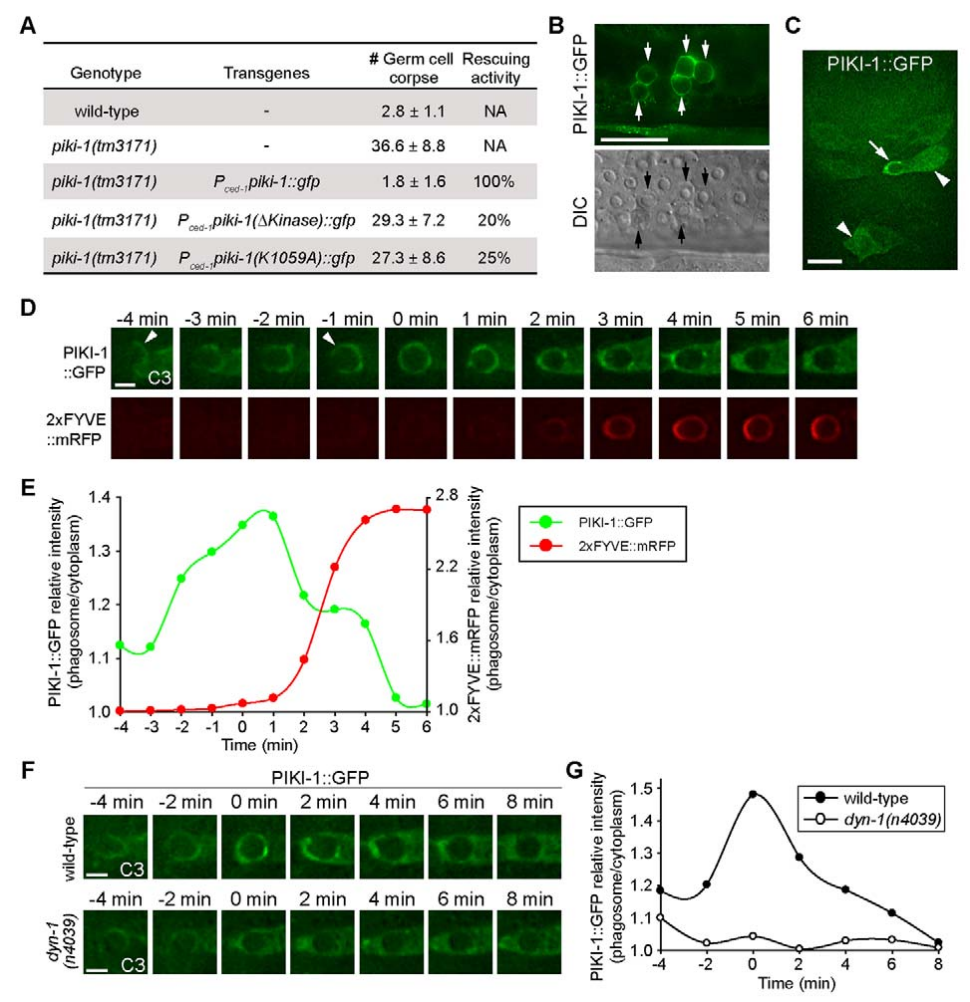
PIKI-1 is recruited onto pseudopods and nascent phagosomes during the removal of apoptotic cells. (A) The rescuing activity of wild-type and mutant PIKI-1::GFP expressed under the control of P*_ced-1_*. The numbers of germ cell corpses in the gonads of adult hermaphrodites carrying particular transgenes were scored at 48 h post-L4 stage. Data are presented as mean ± standard deviation. Fifteen animals were scored for each genotype. Rescuing activity was calculated as [(No_mut_−No_(mut+transgene)_)/(No_mut_−No_wt_)]×100%. No, mean number of germ-cell corpses. (B) Epifluorescence and DIC images of part of the gonad in a wild-type hermaphrodites expressing P*_ced-1_piki-1::gfp*. Arrows indicate phagosomes labeled with PIKI-1::GFP. Scale bars, 20 µm. (C) An epifluorescence image of the ventral surface of a ∼330-min-stage wild-type embryo expressing P*_ced-1_ piki-1::gfp*. An arrow indicates a nascent phagosome labeled by PIKI-1::GPF. Two arrowheads mark two cells that clearly display the cytoplasmic localization pattern of PIKI-1::GFP. Scale bars, 5 µm. (D) Time-lapse images showing the recruitment of PIKI-1::GFP to the extending pseudopods and a nascent C3 phagosome, followed by the rapid production of phagosomal PtdIns(3)P in a wild-type embryo expressing P*_ced-1_ piki-1::gfp* and P*_ced-1_ 2x*FYVE*::mrfp1*. Arrowheads indicate the extending pseudopods. “0 min” represents the time point when engulfment is just completed. Scale bars, 2 µm. Additional examples are shown in Figure S8. (E) The relative PIKI-1 and PtdIns(3)P signal intensity, represented as the ratio of fluorescent signal intensity on the surface of phagosomes to that in the nearby cytoplasm of the host cell, were measured from images in (D) and plotted over time. (F) Time-lapse images showing dynamic localization of PIKI-1::GFP on the extending pseudopods and the nascent C3 phagosome in a wild-type embryo and the lack of the localization in a *dyn-1(n4039)* mutant embryo. Scale bar, 2 µm. (G) The relative PIKI-1 signal intensity, represented as the ratio of PIKI-1::GFP signal intensity on the surface of phagosomes to that in the nearby cytoplasm of the host cell, was measured from images in (F) and plotted over time.

To examine the subcellular localization of PIKI-1, in particular to determine whether PIKI-1 acts on nascent phagosomes to produce PtdIns(3)P, we characterized a PIKI-1::GFP reporter expressed in engulfing cells under the control of P*_ced-1_*. P*_ced-1_piki-1::gfp* fully rescued the Ced phenotype of *piki-1* mutants (Figure 6A), indicating that the function of PIKI-1 in engulfing cells was sufficient for driving phagosome maturation. *piki-1*-expression constructs lacking either the entire PI kinase domain (Figure 2A) or carrying a mutation (K1059A) that disrupted ATP-binding (Figure S9) [39] lost most of the rescuing activity (Figure 6A), indicating that the PtdIns(3)P-production activity of PIKI-1 is essential.

In both embryos and the gonads of adult hermaphrodites, PIKI-1::GFP was primarily localized to the cytoplasm and was enriched on phagosomal surfaces (Figure 6B–C). During cell-corpse removal, PIKI-1::GFP was first detected on extending pseudopods and subsequently further enriched on nascent phagosomes, prior to the rapid appearance of the bright PtdIns(3)P signal (Figures 6D–E, S8). PIKI-1::GFP remained on phagosomal surfaces for ∼6 min before dissociating from phagosomes (Figures 6D–E, S8). This dynamic phagosomal enrichment pattern is consistent with PIKI-1's role in producing the initial PtdIns(3)P molecules on nascent phagosomes (Figure 5H).

The large GTPase DYN-1 plays an essential role in the production of phagosomal PtdIns(3)P [8],[13]. To determine whether DYN-1 plays this role through regulating PIKI-1, we examined the phagosomal localization of PIKI-1 in *dyn-1(n4039)* null mutant embryos. We failed to observe any significant enrichment of PIKI-1::GFP on the surfaces of either the extending pseudopods or nascent phagosomes (Figure 6F–G). This result indicates that DYN-1 triggers PtdIns(3)P production by recruiting PIKI-1 to phagosomes.

### MTM-1 Down-Regulates Phagosomal PtdIns(3)P, an Event Critical for Phagosome Maturation

In wild-type embryos, despite the sequential and combined activities of PIKI-1 and VPS-34, an obvious temporal gap, during which PtdIns(3)P is almost undetectable on phagosomes, was observed between the two PtdIns(3)P peaks (Figures 5A and S6F). In particular, the quickly vanishing PtdIns(3)P signal on phagosomes in *vps-34* mutants suggests the existence of a previously unidentified activity that antagonizes PI3Ks by removing PtdIns(3)P from phagosomes.

**Figure 7:**
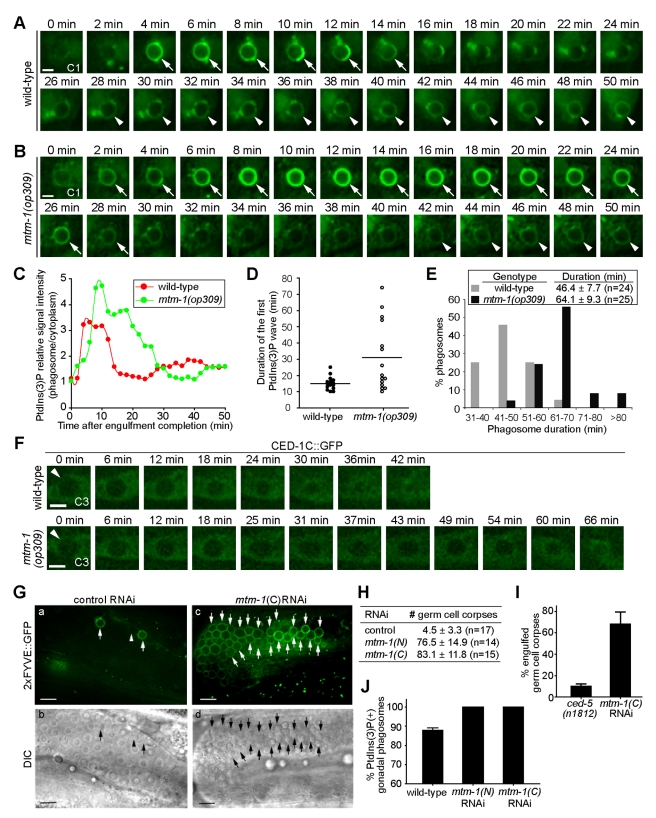
MTM-1 promotes the turnover of phagosomal PtdIns(3)P and is essential for phagosome maturation. (A–B) The temporal presentation patterns of PtdIns(3)P on one C1 phagosome in each of a wild-type (A) or *mtm-1(op309)* mutant (B) embryo were monitored by 2xFYVE::GFP. “0 min” is when engulfment is completed. Arrows and arrowheads indicate the phagosome maturation stages covered by the first and the second waves of PtdIns(3)P on phagosomes, respectively. Scale bars, 2 µm. Additional examples are shown in Figure S10. (C) Quantification of the relative PtdIns(3)P signal intensity (the ratio of GFP signal intensity on the surface of phagosomes versus in the cytoplasm of the host cell) measured from each image in (A–B) and plotted over time. Additional examples are shown in Figure S10. (D) The time-span of the first PtdIns(3)P wave in multiple C1, C2, and C3 phagosomes were displayed in a scatter plot, represented by individual dots. Horizontal lines indicate the average duration of the first PtdIns(3)P wave in each genotype. (E) Histogram distribution of the phagosome duration in embryos determined using the CED-1C::GFP reporter. Duration was defined as a time span during which the diameter of a phagosome was reduced to one-third of the initial size. The mean duration ± SD of each genotype was stated. *n*, the number of C1, C2, and C3 phagosomes measured using time-lapse recording. (F) Time-lapse recording of the degradation process of C3 phagosomes (arrowheads) in a wild-type or a *mtm-1(op309)* mutant embryo expressing P*_ced-1_ ced-1C::gfp*. “0 min” represents the time point when the C3 cell corpse was just fully engulfed, and the newly formed phagosome was recognizable as a dark sphere inside the GFP-labeled cytoplasm of the host cells. Scale bars, 2 µm. (G–J) *mtm-1*(RNAi) phenotypes. RNAi treatments started at L1-stage and animals were analyzed at 48 h after L4 stages. *mtm-1*(N) and *mtm-1*(C) are two non-overlapping RNAi constructs. (G) Epifluorescence (a and c) and DIC (b and d) images of part of gonad arms of wild-type hermaphrodites expressing P*_ced-1_*2xFYVE*::gfp*, after treatment with control or *mtm-1*(C) RNAi. Arrows and arrowheads indicate 2xFYVE::GFP(+) and 2xFYVE::GFP(−) phagosomes, respectively. Dorsal is to the top. Scale bars, 10 µm. (H) The number of germ cell corpses per gonadal arm of wild-type hermaphrodites treated with indicated RNAi. Data were presented as mean ± SD. *n*, number of animals scored. (I) Percentages of engulfed germ cell corpses were scored using GFP::RAB-7 as a phagosomal marker in *ced-5(n1812)* mutant and *mtm-1*(C) RNAi-treated adult hermaphrodites at 48 h post-L4 stage. Data are presented as mean ± SD, obtained from three repeats, each of which scoring 50 phagosomes in the gonad. (J) The percentage of phagosomes labeled with PtdIns(3)P in the gonad of wild-type adult hermaphrodites that expressed P*_ced-1_*2xFYVE*::gfp* and were treated with indicated RNAi. Data are presented as mean ± SD, obtained from three repeats, each of which scoring 50 phagosomes in the gonad.

To identify the negative regulator of phagosomal PtdIns(3)P, we examined the function of *C. elegans* PI 3-phosphatase MTM-1 in regulating PtdIns(3)P dynamics during phagosome maturation. In embryos homozygous for *mtm-1(op309)*, a partial loss-of-function mutation [40], we observed the normal initial appearance of PtdIns(3)P within 2–4 min of phagosome formation. However, the level of the PtdIns(3)P on phagosomes was significantly higher in *mtm-1(op309)* mutant embryos than in wild-type embryos (Figures 7A–C and S10). In addition, the appearance period of PtdIns(3)P on phagosomes was remarkably prolonged: the mean duration of the first PtdIns(3)P wave was more than doubled in *mtm-1(op309)* embryos, with 27% phagosomes lasting longer than 3.6 times of the average length in wild-type embryos (Figures 7A–D and S10). Since MTM-1 is able to dephopshorylate PtdIns(3)P to PtdIns in vitro [40], our observations suggest that MTM-1 might directly dephosphorylate phagosomal PtdIns(3)P.

Previously, whether the down-regulation of phagosomal PtdIns(3)P has any functional significance has not been explored. To address this question, we measured the rate of phagosome maturation in *mtm-1(op309)* embryos, aided by CED-1C (the intracellular domain of CED-1)::GFP expressed in the engulfing cells [8]. CED-1C::GFP, which is evenly distributed in the cytoplasm of host cells, allowed us to detect the GFP(−) phagosomes as dark spheres (Figure 7F) [8]. In *mtm-1(op309)* mutant embryos, phagosome duration was mildly yet significantly (*p* = 2×10^−9^, Student's *t* test) prolonged, with the average duration 38% longer than the wild-type control (Figure 7E–F). This result suggests that the magnified and prolonged action of PtdIns(3)P on phagosomes does not speed up but rather slows down phagosome maturation.

The *mtm-1(op309)* mutation is a missense mutation (G106E) that only partially inactivates MTM-1 [40]. To inactivate MTM-1 more effectively, we performed *mtm-1*(RNAi) (Materials and Methods), which resulted in the accumulation of a large number of germ cell corpses in adult hermaphrodite gonads (Figure 7G–H). This strong Ced phenotype is unlikely a result of an off-target effect of RNAi, since two non-overlapping RNAi constructs, each of which targeting the N- or C-terminal half of *mtm-1* cDNA, respectively, caused the Ced phenotype to the same severity (Figure 7H). Aided by a previously established phagocytosis assay that utilized a GFP::RAB-7 reporter specifically expressed in engulfing cells to distinguish engulfed versus unengulfed cell corpses [21], we further determined that the majority of the germ cell corpses observed in *mtm-1*(RNAi) gonads were engulfed inside gonadal sheath cells (Figure 7I). This phenotype is in direct contrast to that displayed by *ced-5(n1812)* null mutants, which are primarily defective in the engulfment of germ cell corpses (Figure 7I), and indicates that *mtm-1* is primarily required for phagosome maturation.

*mtm-1*(RNAi) increased the percentage of PtdIns(3)P-labeled gonadal phagosomes from 86% to 100% (Figure 7J), again suggesting that PtdIns(3)P is retained on phagosomes once MTM-1 is inactivated. Together, the above results strongly indicate that the turnover of phagosomal PtdIns(3)P is crucial for phagosome maturation.

### MTM-1 Antagonizes the Activities of PIKI-1 and VPS-34 on Phagosomes

**Figure 8:**
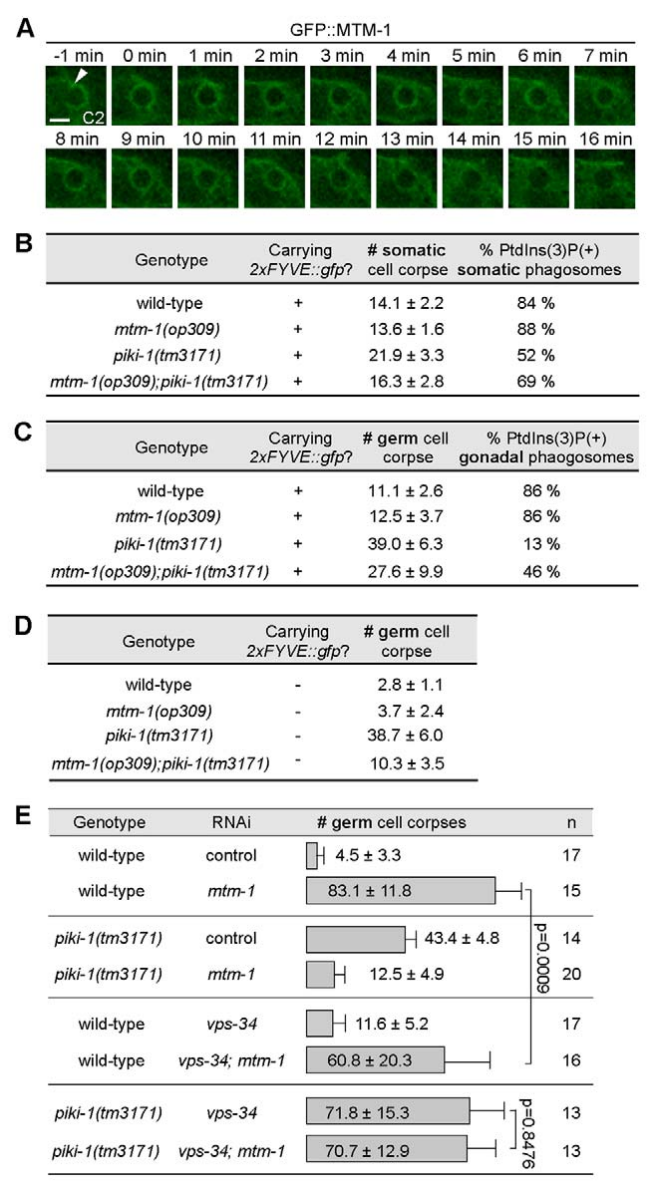
MTM-1 antagonizes the PtdIns(3)P-production activities of PIKI-1 and VPS-34. (A) Time-lapse images showing the dynamic recruitment of GFP::MTM-1 to the phagocytic cup (arrowhead) and subsequently the nascent C2 phagosome in a wild-type embryo expressing P*_ced-1_gfp::mtm-1*. “0 min” represents the time point when engulfment is just completed. Scale bar, 2 µm. (B–C) The numbers of DIC(+) cell corpses and the percentage of 2xFYVE::GFP(+) phagosomes in 2-fold stage embryos (B) or adult hermaphrodites at 48 h after L4 stage (C) that carried transgene P*_ced-1_ 2xFYVE::gfp*. Data are presented as mean ± SD. Fifteen animals were scored for each genotype. (D) The numbers of germ cell corpses in adult hermaphrodites not carrying P*_ced-1_ 2xFYVE::gfp* at 48 h after L4 stage. Data are presented as mean ± SD. Fifteen animals were scored for each genotype. (E) RNAi inactivation of *mtm-1* resulted in severe germ cell corpse removal defect, which was partially rescued by the *piki-1(tm3171)* mutation or *vps-34*(RNAi). RNAi treatments started at L1-stage and animals were scored at 48 h after L4 stages for the numbers of germ cell corpses per gonadal arm. The RNAi constructs are *vps-34*(N) and *mtm-1*(C). Data are presented as mean ± SD. *n*, number of animals scored. *p* values were deduced from independent Student *t*-test.

Using a GFP::MTM-1 reporter expressed specifically in engulfing cells (P*_ced-1_gfp::mtm-1*), we observed the transient association of MTM-1 with the extending pseudopods throughout engulfment and with nascent phagosomes for approximate 13 min (Figure 8A). The phagosome-association pattern of GFP::MTM-1 overlaps with the first PtdIns(3)P wave (Figures 5A and S6F). Furthermore, MTM-1 completely co-localizes with PIKI-1 on phagosomes until PIKI-1 is dissociated from phagosomes (Figure 6D); MTM-1 also overlaps with the PtdIns(3)P-producing activity of VPS-34 during the first but not the second PtdIns(3)P wave (Figure 5H).

To determine the functional relationship between MTM-1 and the two PI3Ks, we first analyzed *mtm-1(op309)*; *piki-1(tm3171)* double mutant animals carrying the PtdIns(3)P reporter 2xFYVE::GFP. In both embryos and adult hermaphrodite gonads, *mtm-1(op309)*; *piki-1(tm3171)* double mutants had significantly fewer numbers of persistent cell corpses than *piki-1(tm3171)* single mutants (Figure 8B–C). Furthermore, the percentage of phagosomes labeled with 2xFVYE::GFP was significantly higher in *mtm-1(op309)*; *piki-1(tm3171)* double mutants than in *piki-1(tm3171)* single mutants (Figure 8B–C). These observations indicate that the partial loss of *mtm-1* function significantly rescued the defects caused by *piki-1(tm3171)* mutation in both PtdIns(3)P production and phagosome maturation. The suppression effect of the Ced phenotype of *piki-1(tm3171)* mutants by the *mtm-1(op309)* mutation became more evident when examined in strains not carrying P*_ced-1_2xFYVE::gfp*, which might compete with endogenous PtdIns(3)P effectors for PtdIns(3)P (Figures 8D, S6, and Text S1). More strikingly, in *piki-1(tm3171)*; *mtm-1*(RNAi) animals, the number of persistent germ cell corpses was drastically reduced to 15% of that observed in *mtm-1*(RNAi) animals and 29% of that in *piki-1(tm3171)* single mutant animals (Figure 8E). Therefore, *piki-1(tm3171)* and *mtm-1*(RNAi), each of which caused a strong Ced phenotype, efficiently rescued each other's defect in phagosome maturation. These results clearly indicate that MTM-1 and PIKI-1 antagonize each other's activity and underscore the importance of a balanced PtdIns(3)P production on phagosomes.

*vps-34*(RNAi) resulted in a relative weak Ced phenotype in the adult hermaphrodite gonads (Figure 8E). *vps-34*(RNAi) modestly reduced the severity of the Ced phenotype of *mtm-1*(RNAi) animals (Figure 8E). The suppression effect of *vps-34*(RNAi) is much weaker than that of *piki-1(tm3171)* mutation, suggesting that PIKI-1 is the major PI3K that counteracts the activity of MTM-1.

Importantly, the strong Ced phenotype caused by the simultaneous inactivation of *piki-1* and *vps-34*, which totally abolished PtdIns(3)P production, was no longer suppressible by *mtm-1*(RNAi) (Figure 8E), clearly demonstrating that MTM-1 specifically targets phagosomal PtdIns(3)P, the products of PIKI-1 and VPS-34.

## Discussion

### A Multi-Component Regulatory System that Establishes the Two-Wave Pattern of PtdIns(3)P on Phagosomes

**Figure 9:**
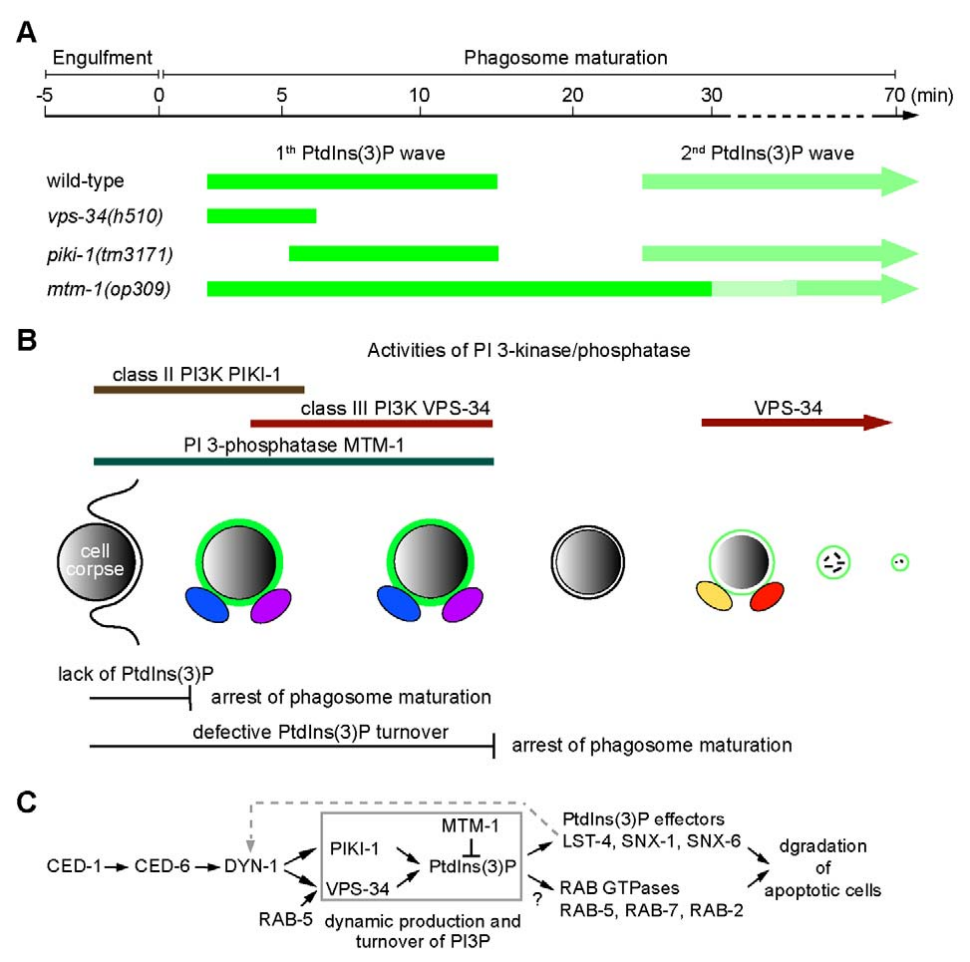
Diagrams depicting the molecular mechanisms that regulate the dynamic presentation of PtdIns(3)P on phagosomes during the degradation of apoptotic cells. (A) Schematic diagram summarizing the oscillation pattern of PtdIns(3)P on phagosomal surfaces in wild-type and different mutant backgrounds. Data represent average values obtained from time-lapse recording of multiple C1, C2, and C3 phagosomes. The light and dark green colors reflect weak and strong PtdIns(3)P signals, respectively. Arrowheads indicate that the end point of the second PtdIns(3)P wave varies, depending on the duration of the phagosome. “0 min” represents the time point when engulfment is just complete. (B) A diagram indicating the functional periods of PIKI-1, VPS-34, and MTM-1 in establishing the biphasic oscillation pattern of PtdIns(3)P on phagosomes, deduced from the PtdIns(3)P localization pattern summarized in (A) and the phagosomal enrichment periods of PIKI-1 and MTM-1. During the gap period, although MTM-1::GFP was not detected on phagosomes, MTM-1 might still display residual activity on phagosomal membranes. The production and elimination of PtdIns(3)P are both essential for phagosome maturation. The rapid production of phagosomal PtdIns(3)P initiates phagosome maturation via recruiting a number of direct or indirect PtdIns(3)P effectors (represented by oval shapes). The subsequent elimination of PtdIns(3)P might enable phagosomes to release the initial set of PtdIns(3)P-effectors and acquire a new set of effectors that function at a later stage of phagosome maturation. (C) Diagram illustrating the PtdIns(3)P-mediated pathway for the degradation of apoptotic cells in *C. elegans*. The phagocytic receptor CED-1 and its adaptor CED-6 recruit the large GTPase DYN-1 to phagosomal surfaces, which in turn promotes the recruitment of PIKI-1 and VPS-34, resulting in the robust production of PtdIns(3)P on nascent phagosomes. RAB-5 is also proposed to facilitate the association of VPS-34 to phagosomes. MTM-1, which is localized on nascent phagosomes, promotes the rapid turnover of PtdIns(3)P. The phagosomal PtdIns(3)P is required for recruiting multiple phagosome maturation factors, including SNX-1, SNX-6, and LST-4/SNX-9, the direct PtdIns(3)P effectors, and small RAB GTPases, which act together to promote phagosome maturation and the degradation of apoptotic cells. LST-4/SNX-9 also acts to stabilize the association of DYN-1 to phagosomes (dashed line). The question mark indicates that the mechanisms utilized by PtdIns(3)P to recruit RAB GTPases are not clear.

Among all PtdIns(3)P-mediated cellular events, phagosome maturation in *C. elegans* provides a unique opportunity for studying the molecular mechanisms and the functional significance of the temporal changes of PtdIns(3)P on intracellular membranes because the dynamics of PtdIns(3)P can be readily monitored, using time-lapse microscopy, on a relatively large object (diameter >2 µm) throughout the entire maturation process (∼50 min or longer). In this report, using genetic and live-cell imaging approaches, we identified a temporal regulation mechanism that programs the two-wave cycling pattern of PtdIns(3)P on phagosomal surfaces (Figure 9). This mechanism is executed by PIKI-1 and VPS-34, two PI 3-kinases that sequentially produce PtdIns(3)P on phagosomes, and MTM-1, a PI 3-phosphatase that dephosphorylates phagosomal PtdIns(3)P. Our findings demonstrate that the precisely regulated production and turnover of phagosomal PtdIns(3)P are both essential for phagosome maturation and the consequential degradation of apoptotic cells.

### The Novel Function of a Class II PI3K in Initiating Phagosome Maturation

The physiological functions of Class II PI3Ks are only starting to be understood. Mammalian Class II PI3Ks are implicated in the production of a dynamic PtdIns(3)P pool on the plasma membrane in response to external stimuli [27],[41]–[43]. Certain Class II PI3Ks are also known to establish various intracellular PtdIns(3)P pools [28],[29],[44]. However, the function of Class II PI3Ks in phagosome maturation was not known. Previously, Class III PI3K Vps34 was thought to be the primary kinase that generated PtdIns(3)P on the surface of phagosomes [12],[13],[16]. Here we identified the novel function of PIKI-1, the only *C. elegans* Class II PI3K, in producing a dynamic pool of PtdIns(3)P on nascent phagosomes and in initiating the degradation of apoptotic cells inside phagosomes. The combined activities of PIKI-1 and VPS-34 account for most if not all PtdIns(3)P molecules on the surface of phagosomes. To our knowledge, this is the first example that two PI3Ks, which belong to different classes, contribute to overlapping pools of PtdIns(3)P on a particular organelle and coordinately regulate the same cellular event.

In *piki-1* and *vps-34* single mutants and *vps-34*; *piki-1* double mutants, defects in PtdIns(3)P-production correlate quantitatively with defects in cell-corpse removal, indicating that the lack of phagosomal PtdIns(3)P is the primary cause for the Ced phenotype. Our observations further indicate that the contribution of PIKI-1 and VPS-34 to phagosome maturation depends on the tissue context: in the adult hermaphrodite gonad, PIKI-1 apparently plays a major role in producing phagosomal PtdIns(3)P.

The accumulation of persistent cell corpses could result from defects in either the engulfment or the degradation of apoptotic cells, two consecutive cellular events executed through different mechanisms [4],[45]. A previous report suggested that *piki-1* mutants were specifically defective in the engulfment of apoptotic cells based on the observation of extra cell corpses in animals, which displayed the unique button-like morphology under DIC microscope [46]. However, persistent cell corpses, which could result from either inefficient engulfment or defective degradation, all display a similar DIC morphology [8],[20],[21],[37]. Thus, the DIC phenotype of cell corpses does not allow the defects in engulfment to be distinguished from that in phagosome maturation. In this study, through both fluorescence and electron microscopy, which are capable of distinguishing unengulfed cell corpses from engulfed ones, we found that PIKI-1 and VPS-34 specifically control the degradation but not the engulfment of apoptotic cells. Our results indicate that the defect of *piki-1* mutants that resulted in the accumulation of cell corpses was misinterpreted previously [46]. Our results are also consistent with the observation that PtdIns(3)P, the molecule produced by PIKI-1 and VPS-34, appears on phagosomal surfaces only after engulfment is complete [7],[8].

Mammals have three Class II PI3K isoforms, which display differential expression patterns (reviewed in [30]). We propose that one or more mammalian Class II PI3Ks might act in phagocytes such as macrophages to promote the degradation of apoptotic cells and other kinds of phagosomal contents such as invading pathogens through producing phagosomal PtdIns(3)P.

### The Differential Roles of PIKI-1 and VPS-34 in Regulating Phagosome Maturation and Other Cellular and Developmental Events

We have found that PIKI-1 and VPS-34 play differential roles in establishing the dynamic PtdIns(3)P pattern on phagosomal surfaces: whereas PIKI-1 initiates the production of PtdIns(3)P on nascent phagosomes, VPS-34 acts to maintain the level of phagosomal PtdIns(3)P for the subsequent period (Figure 9A–B). The functional period of PIKI-1 on phagosomes corresponds to the first half of the initial PtdIns(3)P wave, whereas VPS-34 function is required afterwards, covering the time periods corresponding to the latter part of the initial PtdIns(3)P wave and the entire second wave of PtdIns(3)P (Figure 9A–B). As inactivating either one of the two kinases perturbs the phagosome maturation, we propose that the proper timing of the initial production and the continuous presence of PtdIns(3)P on phagosomes are both crucial for this event.

The molecular mechanism behind the sequential activation of PIKI-1 and VPS-34 is currently under investigation. PIKI-1::GFP is recruited to extending pseudopods and nascent phagosome, prior to the first appearance of PtdIns(3)P, consistent with the role of PIKI-1 in initiating PtdIns(3)P production on nascent phagosomes. The phagosome association of PIKI-1 relies on DYN-1, the key organizer of phagosome maturation events, further suggesting that PIKI-1 might be recruited by DYN-1, directly or indirectly, to its site of action (Figure 9C). This finding places PIKI-1 under the control of the phagosome maturation pathway initiated by the phagocytic receptor CED-1 (Figure 9C) [8]. Mammalian dynamin 2 was reported to directly interact with Vps34, and was proposed to recruit Vps34 to phagosomal surfaces [13]. On the other hand, mammalian Vps34 is one of the Rab5 effectors [2],[47]–[49]. In *C. elegans*, we and others have found that RAB-5 is required for the production of phagosomal PtdIns(3)P (Figure S4 and Text S1) [13]. We have also observed that RAB-5 is recruited to the surfaces of nascent phagosomes prior to the time period when VPS-34's activity is needed for PtdIns(3)P production (Figures 5 and S7). These observations indicate that RAB-5 might also participate in recruiting or activating VPS-34 on phagosomes (Figure 9C).

Vps34 controls multiple membrane trafficking events, some of which, such as endocytosis, are essential for viability (reviewed in [2]). Unlike the *vps-34*(*m* ^−^ *z* ^−^) null mutants, *piki-1* homozygous deletion mutants are viable and fertile, and do not display any obvious endocytosis defects (Figures S1B, S3), suggesting that PIKI-1 specifically functions in promoting phagosome maturation but not in other essential cellular events.

### How Does PtdIns(3)P Perform Its Indispensable Function in Initiating the Degradation of Apoptotic Cells?

Previous studies of Vps34 established the principle that PtdIns(3)P is important for phagosome maturation [12],[13],[16]. We went one step further by developing a strategy (the *vps-34*(*m* ^−^ *z* ^−^); *piki-1* double mutation) that completely blocked phagosomal PtdIns(3)P production, which allowed us to determine quantitatively how fundamental the role of PtdIns(3)P is in initiating phagosome maturation. The *vps-34*(*m* ^−^ *z* ^−^); *piki-1* double mutations resulted in a Ced phenotype as severe as that caused by mutations in known key regulators and executors of cell-corpse degradation, such as mutations in DYN-1 and triple mutations that completely inactivate SNX-9, SNX-1, and SNX-6, three PtdIns(3)P effectors [20],[21]. These observations demonstrate that PtdIns(3)P is absolutely required for the degradation of apoptotic cells during animal development, rather than acting as one of multiple parallel contributing factors.

VPS-34 has been implicated in the recruitment of RAB-5 and RAB-7 to phagosomes [13]. We have revealed that the absence of phagosomal PtdIns(3)P prevents the phagosomal recruitment of SNX-1 and LST-4/SNX-9 as well as three Rab GTPases, RAB-5, RAB-2, and RAB-7. These proteins play important roles in facilitating the fusion of particular types of intracellular organelles such as endosomes and lysosomes to phagosomes. [8],[13],[21],[38],[50]. Furthermore, LST-4/SNX-9 also helps stabilize the association of DYN-1 with phagosomes [21]. These findings further illuminate the molecular mechanisms used by PtdIns(3)P to initiate phagosome maturation.

### The Novel Physiological Role of PtdIns(3)P Down-Regulation for Phagosome Maturation

MTM1, a member of the myotubularin PI phosphatase family, helps maintain the optimal level of PtdIns(3)P on the surface of multiple kinds of intracellular membranes by converting PtdIns(3)P to PtdIns (reviewed in [29],[31]). Here we have revealed that MTM-1 controls cell-corpse degradation through regulating the turnover of phagosomal PtdIns(3)P (Figure 9). That MTM-1 function is important for the degradation of apoptotic-cell has also been reported independently [40].

The *mtm-1(op309)* mutation slows down phagosome maturation in embryos. Likewise, *mtm-1*(RNAi) results in a dramatic phagosome maturation arrest in adult gonads. These results demonstrate that despite being a negative regulator of PtdIns(3)P, MTM-1 is an essential positive regulator for phagosome maturation. Furthermore, the *piki-1* deletion mutation and *mtm-1*(RNAi) mutually suppresses the phagosome maturation defects of each other. In addition, *vps-34*(RNAi) partially suppresses the phagosome maturation defects caused by *mtm-1*(RNAi). On the contrary, *mtm-1*(RNAi)'*s* suppression effect no longer exists in *piki-1*; *vps-34*(RNAi) double mutant backgrounds. Together, these observations demonstrate that MTM-1 directly antagonizes the function of the two PI3Ks on phagosomes by dephosphorylating their product, PtdIns(3)P. The association of MTM-1 with the extending pseudopods and nascent phagosomes correlates, completely and partially, with the PtdIns(3)P-producing activities of PIKI-1 and VPS-34 on phagosomes, respectively, further supporting the model that MTM-1 antagonizes the activities of PIKI-1 and VPS-34 and results in the gap period between the two PtdIns(3)P waves on phagosomes (Figure 9A and B). During the PtdIns(3)P(−) gap period, a low-level activity of MTM-1 might remain on phagosomal surfaces despite that the enrichment of GFP::MTM-1 on phagosomal surfaces is below the detection capacity of our fluorescence microscope.

We propose that the prompt dephosphorylation of phagosomal PtdIns(3)P might be critical for the timely dissociation of certain initial phagosome maturation factors from phagosomes as well as the subsequent association of certain other, perhaps yet-to-be identified maturation factors that act at later stages of phagosome maturation (Figure 9B). The dynamic oscillation of phagosomal PtdIns(3)P thus would enable phagosomes to interact with various signaling modules and intracellular organelles in sequence, and promote the step-wise progression of phagosome maturation (Figure 9B). It would be of general interest to identify each of the time-sensitive phagosome maturation factors subject to this regulation.

Recently, MTM-1 was reported to negatively regulate the engulfment of apoptotic cells, one step prior to the degradation of apoptotic cells [40],[46]. Zou et al. [46] proposed that MTM-1 negatively regulates the level of PtdIns(3)P on the plasma membrane produced by VPS-34 and PIKI-1 during engulfment, based on the observations that inactivating *piki-1* and *vps-34* resulted in the accumulation of persistent cell corpses and that the ability of MTM-1 to suppress the engulfment defect of *ced-1* and *ced-6* mutants relied on the functions of PIKI-1 and VPS-34. Zou et al.'s work identified the antagonizing genetic interaction between MTM-1 and the two PI3Ks during cell-corpse removal [46]. However, the dynamic PtdIns(3)P appearance pattern and the timing of PIKI-1 and VPS-34 functions do not support a role of PtdIn(3)P in engulfment. In both mammalian cells and *C. elegans*, the production of PtdIns(3)P is initiated on nascent phagosomes after the completion of engulfment, and PtdIns(3)P has not been detected on the extending pseudopods [7],[8],[10]–[13]. In fact, the experimental results reported here revealed that a precisely regulated system, composed of MTM-1, PIKI-1, and VPS-34, promotes the degradation but not the engulfment of apoptotic cells, through an accurate temporal regulation of phagosomal PtdIns(3)P.

On the other hand, Neukomm et al. (2011), who independently identified the function of MTM-1 as a negative regulator of cell-corpse engulfment, proposed that during engulfment, MTM-1 performs its function through dephosphorylating another substrate, PtdIns(3,5)P_2_ [31],[40]. *C. elegans* MTM-1 might play opposite roles in engulfment and degradation, two consecutive steps of apoptotic-cell removal, by down-regulating different phosphoinositide species.

This study, together with a few recent reports, have highlighted the physiological importance of the antagonizing activities of PI kinases and phosphatases in the regulation of PtdIns(3)P dynamics on various intracellular membranes [29],[51]. Like the cycling of small GTPases between GDP- and GTP-bound states and the reversible phosphorylation of key protein factors, the reversible phosphorylation of various phophoinositide species on defined membrane domains is likely to be utilized as a common strategy for driving the progression of multi-step biological events.

## Materials and Methods

### Mutations and Strains

*C. elegans* strains were grown at 20°C as previously described [52]. The N2 Bristol strain was used as the reference wild-type strain. Mutations are described in [53], the Worm Base (www.wormbase.org), and this work, except when noted otherwise: LGI, *vps-34(h510)* [35], *unc-108/rab-2(n3263)* [7], *mtm-1(op309)* [40]; LGII, *rab-7*(*ok511*) [8]; LGIV, *ced-5*(*n1812*); LGV, *unc-76*(*e911*); LGX, *piki-1(tm3171)*, *piki-1(ok2346)*, *dyn-1(n4039)* [20]. Strains carrying the *piki-1(tm3171)* and *piki-1(ok2346)* alleles were characterized after being out-crossed for four times. Transgenic worms were generated by microinjection as previously described [54]. Plasmids were co-injected with a marker pUNC-76 [*unc-76(+)*] [55] into *unc-76*(*e911*) mutant adult hermaphrodites and transgenic animals were identified as non-Unc animals. Transgenes are maintained in animals as extra-chromosomal arrays and, when necessary, introduced into different genetic backgrounds by crosses.

### Plasmid Construction

The *piki-1* cDNA was amplified from a mixed-stage *C. elegans* cDNA library (Z. Zhou and H. R. Horvitz, unpublished results) using polymerase chain reaction (PCR). P*_ced-1_piki-1::gfp* was constructed by cloning the *piki-1* cDNA into the multi-cloning sites of pZZ829, a plasmid carrying *P_ced-1_*, a 3′ *gfp* tag, and the *unc-54* 3′ UTR [20]. The overlap extension PCR method [56] was used to delete the DNA sequence encoding the PI kinase domain from P*_ced-1_piki-1::gfp* and generate P*_ced-1_piki-1(Δkinase)::gfp*. To generate P*_ced-1_piki-1(K1059A)::gfp*, the K1059A mutation was introduced into P*_ced-1_piki-1::gfp* using the QuickChange Site-directed Mutagenesis Kit (Stratagene). To make P*_ced-1_gfp::mtm-1*, *mtm-1* cDNA was amplified from the mixed-stage *C. elegans* cDNA library using PCR and subsequently cloned into pZZ956, a plasmid carrying *P_ced-1_*, a 5′ *gfp* tag, and the *unc-54* 3′ UTR.

### Nomarski DIC Microscopy to Identify Cell Corpses

DIC microscopy was performed with an Axionplan 2 compound microscope (Carl Zeiss, Inc.) equipped with Nomarski DIC optics, a digital camera (AxioCam MRm; Carl Zeiss) and imaging software (AxioVision; Carl Zeiss). Worms were immobilized with 25 mM NaN_3_, mounted on 4% agarose pads, and observed under DIC microscopy. Somatic embryonic cell corpses were scored in the embryos at different developmental stages as described by [33]. Germ cell corpses were scored in one of the two gonadal arms of synchronized adult hermaphrodites at indicated time-points post-L4 stages [20].

### Fluorescence Microscopy and Time-Lapse Recording

An Olympus IX70-Applied Precision DeltaVision microscope equipped with standard epifluorescent filter sets and Photometris Coolsnap digital camera was used to capture fluorescence images, which were deconvolved and processed by the Applied Precision SoftWoRx software. To score the numbers of somatic or germ phagosomes labeled by GFP or mRFP reporters on their surfaces, z-sections of DIC and fluorescent images spanning the entire depth of embryos or adult gonads, respectively, were captured and analyzed [8]. To monitor the dynamic subcellular localization pattern of various GFP reporters during the engulfment and degradation of cell corpses C1, C2, and C3, the procedure followed an established protocol [33]. Briefly, time-lapse recording started at 310–320 min post-first cleavage and lasted for 60–120 min at 1 or 2 min intervals. At each time point, 10–15 serial Z-sections at a thickness of 0.5 µm were recorded, starting from the ventral surface of embryos. Signs such as embryo elongation and movement were closely monitored to ensure that the embryo being recorded developed normally. Whenever necessary, fluorescence signal intensity was measured and images were analyzed using the ImageJ software [33]. To compare fluorescence signal intensity on phagosomes in wild-type or mutant embryos, the images were captured using the same microscopic parameters, processed, and analyzed using the same procedures.

### RNA Interference (RNAi)

RNAi experiments were performed using feeding protocol as previously described [57]. In brief, mid-L4-stage hermaphrodites (for *vps-34*(RNAi) experiments in Figures 1 and S1) or synchronized L1-stage hermaphrodites (for *mtm-1*(RNAi) and *vps-34*(RNAi) experiments in Figure 8) were transferred to RNAi plates that contained *E. coli* strain HT115 transformed with RNAi constructs. For RNAi experiments starting at L1 stage, mid-L4-stage animals were retransferred to fresh RNAi plates. 48 h after L4 stage, the number of germ cell corpses per gonad arm were scored under DIC and fluorescent microscopy. The RNAi-by-feeding vector pPD129.36 [58] was used as a negative control for RNAi. The RNAi feeding constructs for *vps-34* were generated by cloning PCR-amplified cDNA fragments (*vps-34(N)* and *vps-34(C)*) corresponding to the N- and C-terminal halves of VPS-34 into vector pPD129.36 using the following primers: *vps-34*(N) (5′-ttgggaacacgaggatgatg-3′ and 5′-gttcaggatcagctacacag-3′), *vps-34*(C) (5′-taaaggagtccatc-3′ and 5′-ttgtcaagatgacgatcacc-3′). The RNAi feeding constructs for *mtm-1* were generated by cloning PCR-amplified cDNA fragments corresponding to the N- and C-terminal halves of MTM-1 into vector pPD129.36 using the following primers: *mtm-1*(N) (5′-gcgccccgggatggattcacaatttattg-3′ and 5′-atatcccgggctcgccgagcttctttac-3′), *mtm-1*(C) (5′-aaaggaaattttcagccaatgtt-3′ and 5′-tgcacataaagaaagcaaaatga-3′).

### Transmission Electron Microscopy

Electron microscopy was performed as previously described [59]. Briefly, 2-d-old adult worms were fixed in 0.67% gluteraldehyde and 0.67% Osmium tetroxide in 10 mM HEPES buffer with microwave fixation 2 times at 90 W for 2 min/ON, 2 min/OFF, 2 min/ON. Worms were cut at the vulva and fixed for 1 h on ice. Worms were then fixed in 2% osmium tetroxide in 10 mM HEPES by microwaving two times at 90 W or 2 min/ON, 2 min/OFF, 2 min/ON and were subsequently incubated on ice for 3 h. Further processing was performed as previously described [59]. Standard procedures were used to generate and stain 50–60 nm sections. Electron microscopy was performed and images were captured with a digital camera.

## Supporting Information

Figure S1 The phenotypes of *vps-34*(RNAi) and *vps-34*(m^−^z^−^) animals (related to Figure 1). (A) The phagosome maturation defect in *vps-34*(RNAi)-treated worms could be further enhanced by overexpressing 2xFYVE::GFP reporter in engulfing cells. The numbers of germ cell corpses were scored in adult hermaphrodites treated with *vps-34* RNAi for 48 h from L4 stage. Animals either carried or did not carry the P*_ced-1_*2xFYVE*::gfp* transgenic array, as indicated. *vps-34*(N) and *vps-34*(C) are two independent RNAi feeding constructs targeting different regions of *vps-34* coding sequence. Data are presented as mean ± standard deviation (SD). *n*, number of animals scored. (B) The depletion of both maternal and zygotic *vps-34* products (indicated by m^−^z^−^) resulted in the arrest of animal development at earlier stages than solely depleting zygotic *vps-34* products (indicated by m^+^z^−^). The percentages of animals arrested during embryogenesis or at each of the four larval stages were shown as bar graph.
(TIF)

Figure S2 Gene structure of *piki-1* and phenotype of two *piki-1* deletion mutants (related to Figure 1). (A) Gene structures and locations of two *piki-1* deletion alleles. The coding and non-coding regions of exons are shown as black and open boxes, respectively. Introns are indicated by thin lines between exons. The bars underneath the gene indicate the genomic regions that were removed in each deletion allele. Numbers represent nucleotide numbers. (B) Domain structure of wild-type PIKI-1 and the predicted truncated forms of PIKI-1 encoded by two deletion alleles. Shaded domains indicate the domains, part of which were deleted by each deletion allele. UIM, Ubiquitin-interacting motif; RBD, Ras-binding domain; C2. Protein kinase C conserved region 2; PIK, Phosphoinositide 3-kinase, accessory domain; Kinase, Phosphoinositide 3-kinase, catalytic domain; PX, PhoX homologous domain. (C) DIC images of part of gonadal arms of adult hermaphrodites at 48 h after L4 stages. Arrows indicate germ cell corpses. Dorsal is to the top. Scale bars, 20 µm. (D) The numbers of germ cell corpses in adult hermaphrodites of different ages are displayed in a bar graph. Germ cell corpses were scored every 12 h after the L4 stage. Fifteen animals were scored for each data point. Data are presented as mean ± SD. (E) The numbers of somatic cell corpses at different embryonic stages are displayed in a bar graph. At least 15 embryos were scored for each data point. Data are presented as mean ± SD.
(TIF)

Figure S3 *piki-1(tm3171)* mutants are normal for endocytosis. (A–B) The endocytosis (A) and redistribution (B) of yolk is normal in *piki-1(tm3171)* mutant oocytes and embryos, respectively, monitored by the YP170::GFP reporter. (A) Epifluorescence (a–b) and DIC (c–d) images of adult hermaphrodite gonad in wild-type (a, c) and *piki-1(tm3171)* (b, d) adult hermaphrodites. Filled arrows indicate oocytes filled with YP170::GFP, filled arrowheads indicate embryos, and open arrows indicate spermathecae. Scale bars, 10 µm. (B) Epifluorescence and DIC images of wild-type (a–f) and *piki-1(tm3171)* (g–m) embryos at different stages (labeled as min post the first cleavage). Arrows indicate intestinal precursor cells, which are enriched with YP170::GFP. Solid lines indicate the head region in 450-min-stage embryos, from which the YP170::GFP is depleted. Scale bars, 10 µm. (C–D) The endocytosis of ssGFP (secreted GFP) by coelomocytes is normal in *piki-1(tm3171)* mutant adults, monitored with the P*_myo-3_*::ssGFP reporter. (C) Epifluorescence (a–b) and DIC (c–d) images of wild-type and *piki-1(tm3171)* mutant adults. Arrows indicate coelomocytes. Scale bars, 20 µm. (D) Efficiency of endocytosis of ssGFP by coelomocytes in wild-type and *piki-1(tm3171)* mutant adults. *n*, number of animals analyzed.
(TIF)

Figure S4 RAB-5, but not RAB-2 or RAB-7, is required for the production of PtdIns(3)P on phagosomes (related to Figure 4E). DIC (a–d) and epifluorescence (e–h) images of part of gonad arms in adult hermaphrodites expressing P*_ced-1_*2xFYVE*::gfp* in gonadal sheath cells. Animals were analyzed at 48 h after L4 stages. Arrows and arrowheads indicate 2xFYVE::GFP(+) and 2xFYVE::GFP(−) phagosomes, respectively. One open arrowhead in (b) indicates a blob of unengulfed yolk resulted from defects in endocytosis caused by *rab-5*(RNAi). Dorsal is to the top. Scale bars, 20 µm.
(TIF)

Figure S5 The overexpression of SNX-1 or LST-4 does not affect phagosome maturation (related to Figure 3). (A) The numbers of somatic cell corpses scored at different embryonic stages in wild-type embryos carrying indicated transgenes. At least 20 embryos were scored for each data point. Data are presented as mean ± SD. *p<0.05 by independent Student's *t*-test. “NS” indicates non-significant differences. (B) Histogram distribution of the phagosome duration in embryos expressing indicated transgenes. The duration of phagosomes also displayed as mean ± sd. *n*, the number of C1, C2, and C3 phagosomes measured.
(TIF)

Figure S6 The sequestration effect of 2xFYVE::GFP reporter on phagosomal PtdIns(3)P is dependent on the expression level of the transgene (related to Figure 5). (A) The numbers of somatic cell corpses scored at different embryonic stages in wild-type embryos carrying indicated transgenes. High and low level of 2xFYVE::GFP expression was achieved by injecting worms with 20 ng/µl and 1 ng/µl plasmids, respectively. At least 20 embryos were scored for each data point. Data are presented as mean ± SD. *p<0.05, independent Student's *t*-test. “NS” indicates non-significant differences. (B) A diagram showing that the over-expressed 2xFYVE::GFP molecules may compete with endogenous PtdIns(3)P effectors for the interaction with phagosomal PtdIns(3)P. (C–D) Time-lapse images of the degradation of C3 phagosomes in wild-type embryos expressing transgenes P*_ced-1_* CED-1C*::gfp*(C) or P*_ced-1_* 2xFYVE*::gfp* at a relatively high level. (D) “0 min” represents the time point when a C3 cell corpse was just fully internalized by its engulfing cell, ABplaapppp, and the newly formed phagosome was recognizable as a dark sphere inside GFP(+) engulfing cell. Anterior is to the top. Ventral faces readers. Arrows indicate cell corpses C1, C2, or C3; arrowheads indicate their corresponding engulfing cells; open arrowheads in D(d) indicate nuclei. Scale bars, 5 µm. (E–F) Time-lapse images of the degradation of C3 phagosomes in wild-type embryos expressing transgenes P*_ced-1_* CED-1C*::gfp* (E) or P*_ced-1_* 2xFYVE*::gfp* at low level (F). “0 min” is when engulfment just completed. Scale bars, 2 µm.
(TIF)

Figure S7 The timing of the transient enrichment of RAB-5 on nascent phagosomes is not affected by the expression of the 2xFYVE::GFP reporter. (A–B) Time-lapse recording of the dynamic phagosomal localization of GFP::RAB-5 on C2 phagosomes in wild-type embryos that expressed GFP::RAB-5 alone (A) or that co-expressed GFP::RAB-5 and high level of 2xFYVE::mRFP (B). “0 min” represents the time point when engulfment is just complete. Scale bars, 2 µm. (C) The relative GFP::RAB-5 signal intensity, represented as the ratio of GFP::RAB-5 signal intensity on the surface of phagosomes to that in the nearby cytoplasm of the host cell, was measured from images in (A–B) and plotted over time. (D) Quantification of the timing of the initial appearance of RAB-5 on phagosomes and the duration of RAB-5 in association with phagosomes. Data are presented as mean ± SD. *n*, the number of C1, C2, and C3 phagosomes analyzed.
(TIF)

Figure S8 Three additional examples of the recruitment of PIKI-1 to the extending pseudopods and nascent phagosomes prior to the production of phagosomal PtdIns(3)P (related to Figure 6). Three time-lapse image series of the C3 phagosome in wild-type embryos co-expressing PIKI-1::GFP and 2xFYVE::mRFP. “0 min” represents the time point when engulfment is just complete. Arrowheads indicate the extending pseudopods. Scale bars, 2 µm.
(TIF)

Figure S9 Sequence alignment of the kinase domain of class II PI 3-kinases that include *C. elegans* PIKI-1, *H. sapiens* PI3KC2α, and *D. melanogaster* PI3K68D (related to Figure 6). Lysine 1059, the conserved residue in the ATP binding motif, which was mutated in P*_ced-1_piki-1(K1059A)::gfp*, is labeled by a red frame.
(TIF)

Figure S10 Additional examples of the enhanced and prolonged PtdIns(3)P signal on the phagosomes in *mtm-1(op309)* mutants (related to Figure 7). (A–B) The temporal presentation patterns of PtdIns(3)P on C2 phagosomes in a wild-type (A) or a *mtm-1(op309)* mutant (B) embryo were monitored by 2xFYVE::GFP. “0 min” is the time point when engulfment is just completed. Arrows and arrowheads indicate the phagosome maturation stages covered by the first and the second waves of PtdIns(3)P on phagosomes, respectively. Scale bars, 2 µm. (C) The relative PtdIns(3)P signal intensity, represented as the ratio of 2xFYVE::GFP signal intensity on the surface of phagosomes to that in the nearby cytoplasm of the host cell, was measured from images in (A–B) and plotted over time.
(TIF)

Text S1 Supplemental text includes the following sections. Depletion of maternal and zygotic *vps-34* gene products; characterization of *piki-1* deletion alleles; normal endocytosis in *piki-1* mutants; the requirement of RAB-5 for phagosomal PtdIns(3)P production; and the characterization of the effect of SNX-1::GFP, LST-4::GFP and 2xFYVE::GFP overexpression on phagosome maturation.
(DOCX)

## Abbreviations

Ced: cell-corpse removal defective
DIC: Differential Interference Contrast
PI3K: PI 3-kinase
PtdIns: phosphatidylinositol
PtdIns(3)P: Phosphatidylinositol 3-phosphate
RNAi: RNA interference
TEM: transmission electron microscopy
