# Supplementary figures and images for: Two PI 3-Kinases and One PI 3-Phosphatase Together Establish the Cyclic Waves of Phagosomal PtdIns(3)P Critical for the Degradation of Apoptotic Cells
Source: PLoS Biol. 2012 Jan 17;10(1):e1001245. doi: 10.1371/journal.pbio.1001245 (PMC3260314; doi:10.1371/journal.pbio.1001245)

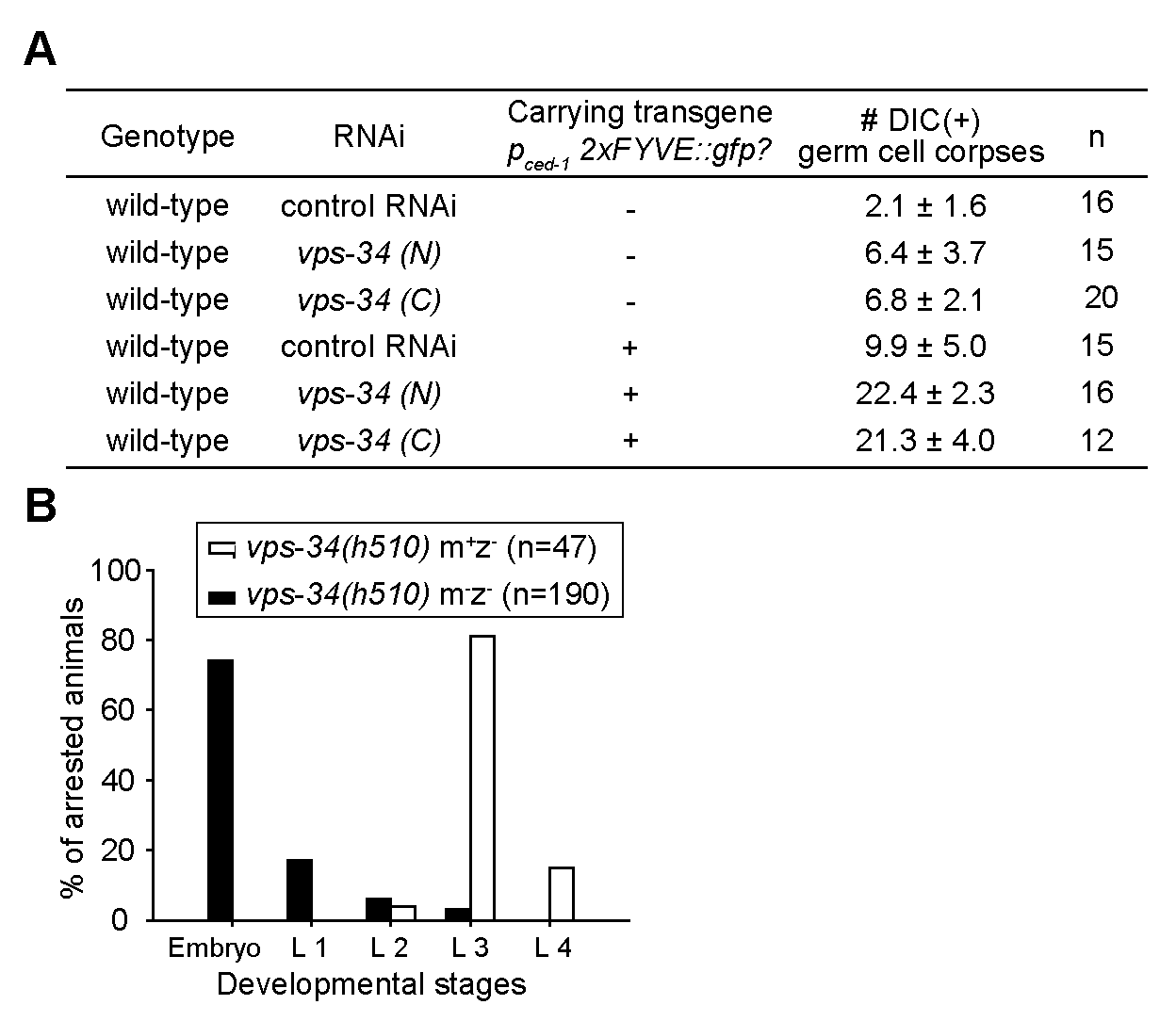

Supplement: Figure S1 — The phenotypes of vps-34(RNAi) and vps-34(m−z−) animals (related to Figure 1). (A) The phagosome maturation defect in vps-34(RNAi)-treated worms could be further enhanced by overexpressing 2xFYVE::GFP reporter in engulfing cells. The numbers of germ cell corpses were scored in adult hermaphrodites treated with vps-34 RNAi for 48 h from L4 stage. Animals either carried or did not carry the Pced-12xFYVE::gfp transgenic array, as indicated. vps-34(N) and vps-34(C) are two independent RNAi feeding constructs targeting different regions of vps-34 coding sequence. Data are presented as mean ± standard deviation (SD). n, number of animals scored. (B) The depletion of both maternal and zygotic vps-34 products (indicated by m−z−) resulted in the arrest of animal development at earlier stages than solely depleting zygotic vps-34 products (indicated by m+z−). The percentages of animals arrested during embryogenesis or at each of the four larval stages were shown as bar graph. (TIF) [file pbio.1001245.s001.tif]

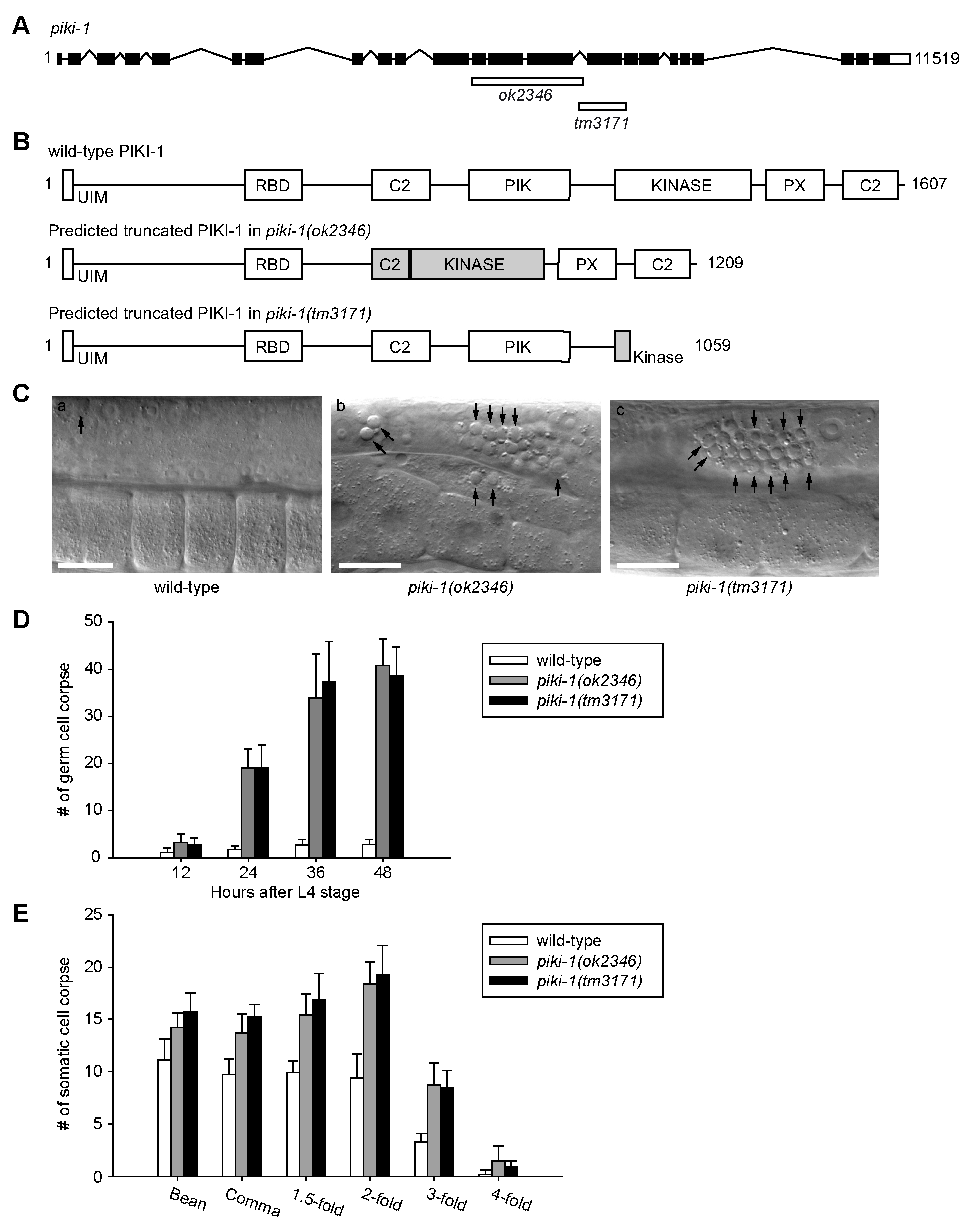

Supplement: Figure S2 — Gene structure of piki-1 and phenotype of two piki-1 deletion mutants (related to Figure 1). (A) Gene structures and locations of two piki-1 deletion alleles. The coding and non-coding regions of exons are shown as black and open boxes, respectively. Introns are indicated by thin lines between exons. The bars underneath the gene indicate the genomic regions that were removed in each deletion allele. Numbers represent nucleotide numbers. (B) Domain structure of wild-type PIKI-1 and the predicted truncated forms of PIKI-1 encoded by two deletion alleles. Shaded domains indicate the domains, part of which were deleted by each deletion allele. UIM, Ubiquitin-interacting motif; RBD, Ras-binding domain; C2. Protein kinase C conserved region 2; PIK, Phosphoinositide 3-kinase, accessory domain; Kinase, Phosphoinositide 3-kinase, catalytic domain; PX, PhoX homologous domain. (C) DIC images of part of gonadal arms of adult hermaphrodites at 48 h after L4 stages. Arrows indicate germ cell corpses. Dorsal is to the top. Scale bars, 20 µm. (D) The numbers of germ cell corpses in adult hermaphrodites of different ages are displayed in a bar graph. Germ cell corpses were scored every 12 h after the L4 stage. Fifteen animals were scored for each data point. Data are presented as mean ± SD. (E) The numbers of somatic cell corpses at different embryonic stages are displayed in a bar graph. At least 15 embryos were scored for each data point. Data are presented as mean ± SD. (TIF) [file pbio.1001245.s002.tif]

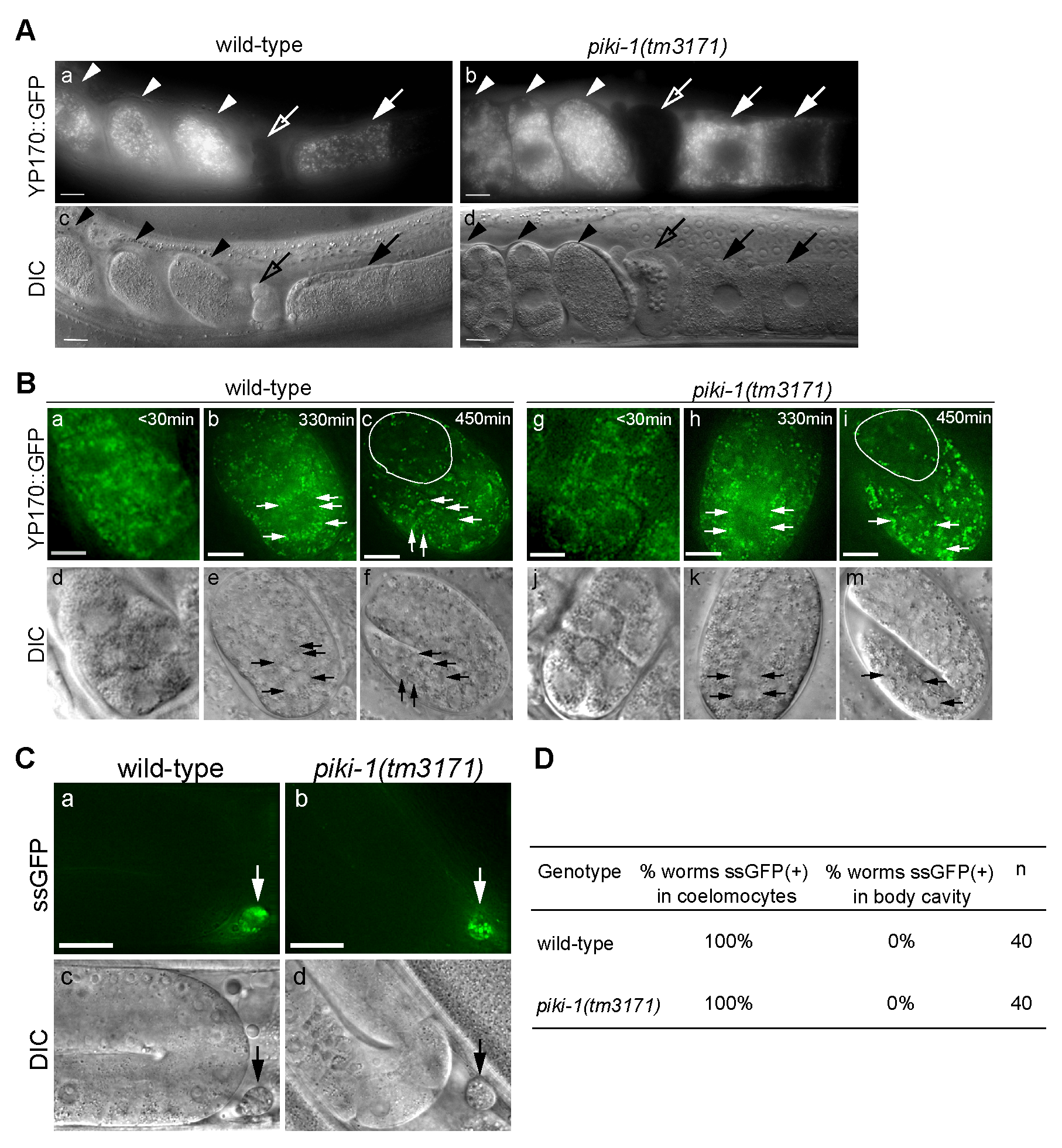

Supplement: Figure S3 — piki-1(tm3171) mutants are normal for endocytosis. (A–B) The endocytosis (A) and redistribution (B) of yolk is normal in piki-1(tm3171) mutant oocytes and embryos, respectively, monitored by the YP170::GFP reporter. (A) Epifluorescence (a–b) and DIC (c–d) images of adult hermaphrodite gonad in wild-type (a, c) and piki-1(tm3171) (b, d) adult hermaphrodites. Filled arrows indicate oocytes filled with YP170::GFP, filled arrowheads indicate embryos, and open arrows indicate spermathecae. Scale bars, 10 µm. (B) Epifluorescence and DIC images of wild-type (a–f) and piki-1(tm3171) (g–m) embryos at different stages (labeled as min post the first cleavage). Arrows indicate intestinal precursor cells, which are enriched with YP170::GFP. Solid lines indicate the head region in 450-min-stage embryos, from which the YP170::GFP is depleted. Scale bars, 10 µm. (C–D) The endocytosis of ssGFP (secreted GFP) by coelomocytes is normal in piki-1(tm3171) mutant adults, monitored with the Pmyo-3::ssGFP reporter. (C) Epifluorescence (a–b) and DIC (c–d) images of wild-type and piki-1(tm3171) mutant adults. Arrows indicate coelomocytes. Scale bars, 20 µm. (D) Efficiency of endocytosis of ssGFP by coelomocytes in wild-type and piki-1(tm3171) mutant adults. n, number of animals analyzed. (TIF) [file pbio.1001245.s003.tif]

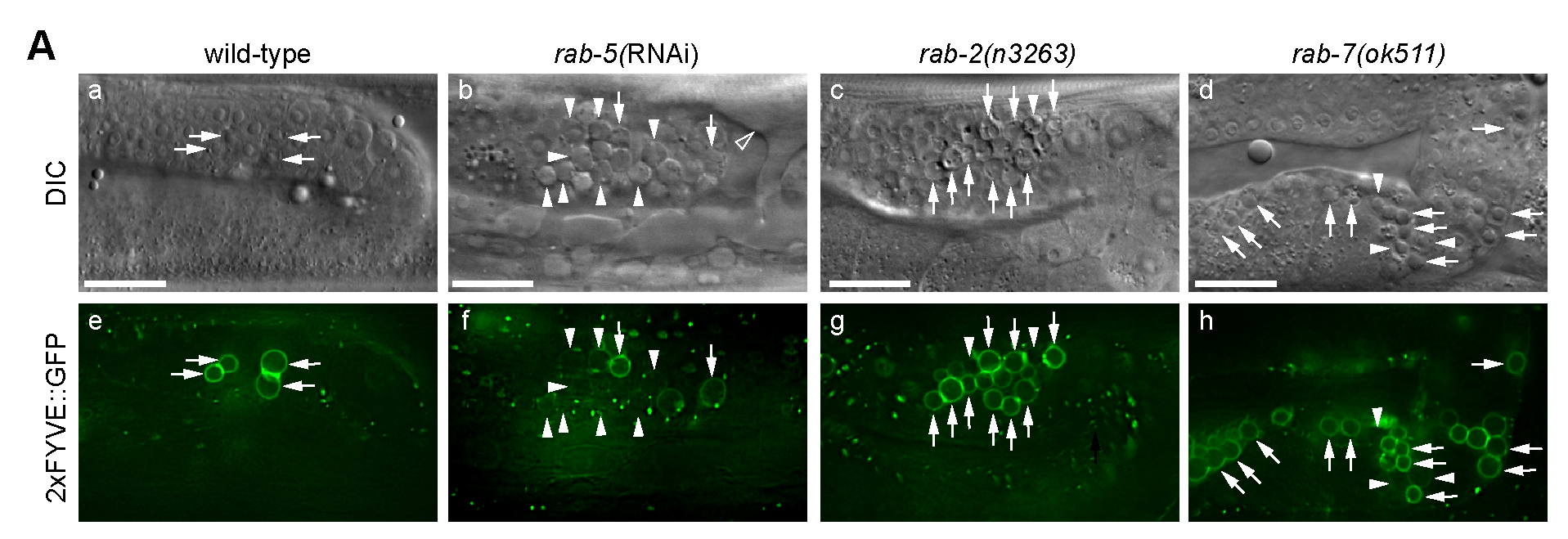

Supplement: Figure S4 — RAB-5, but not RAB-2 or RAB-7, is required for the production of PtdIns(3)P on phagosomes (related to Figure 4E). DIC (a–d) and epifluorescence (e–h) images of part of gonad arms in adult hermaphrodites expressing Pced-12xFYVE::gfp in gonadal sheath cells. Animals were analyzed at 48 h after L4 stages. Arrows and arrowheads indicate 2xFYVE::GFP(+) and 2xFYVE::GFP(−) phagosomes, respectively. One open arrowhead in (b) indicates a blob of unengulfed yolk resulted from defects in endocytosis caused by rab-5(RNAi). Dorsal is to the top. Scale bars, 20 µm. (TIF) [file pbio.1001245.s004.tif]

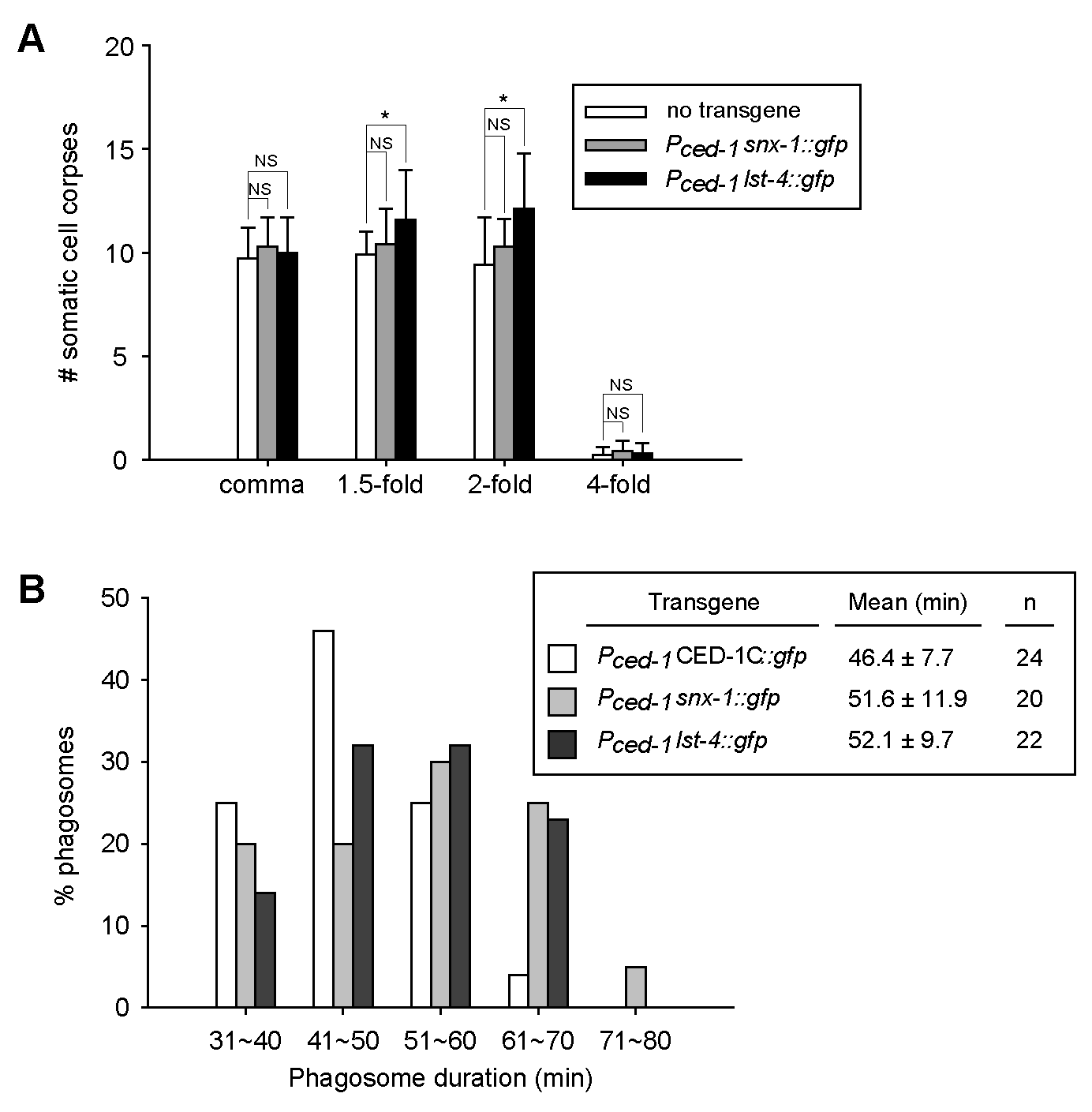

Supplement: Figure S5 — The overexpression of SNX-1 or LST-4 does not affect phagosome maturation (related to Figure 3). (A) The numbers of somatic cell corpses scored at different embryonic stages in wild-type embryos carrying indicated transgenes. At least 20 embryos were scored for each data point. Data are presented as mean ± SD. *p<0.05 by independent Student's t-test. “NS” indicates non-significant differences. (B) Histogram distribution of the phagosome duration in embryos expressing indicated transgenes. The duration of phagosomes also displayed as mean ± sd. n, the number of C1, C2, and C3 phagosomes measured. (TIF) [file pbio.1001245.s005.tif]

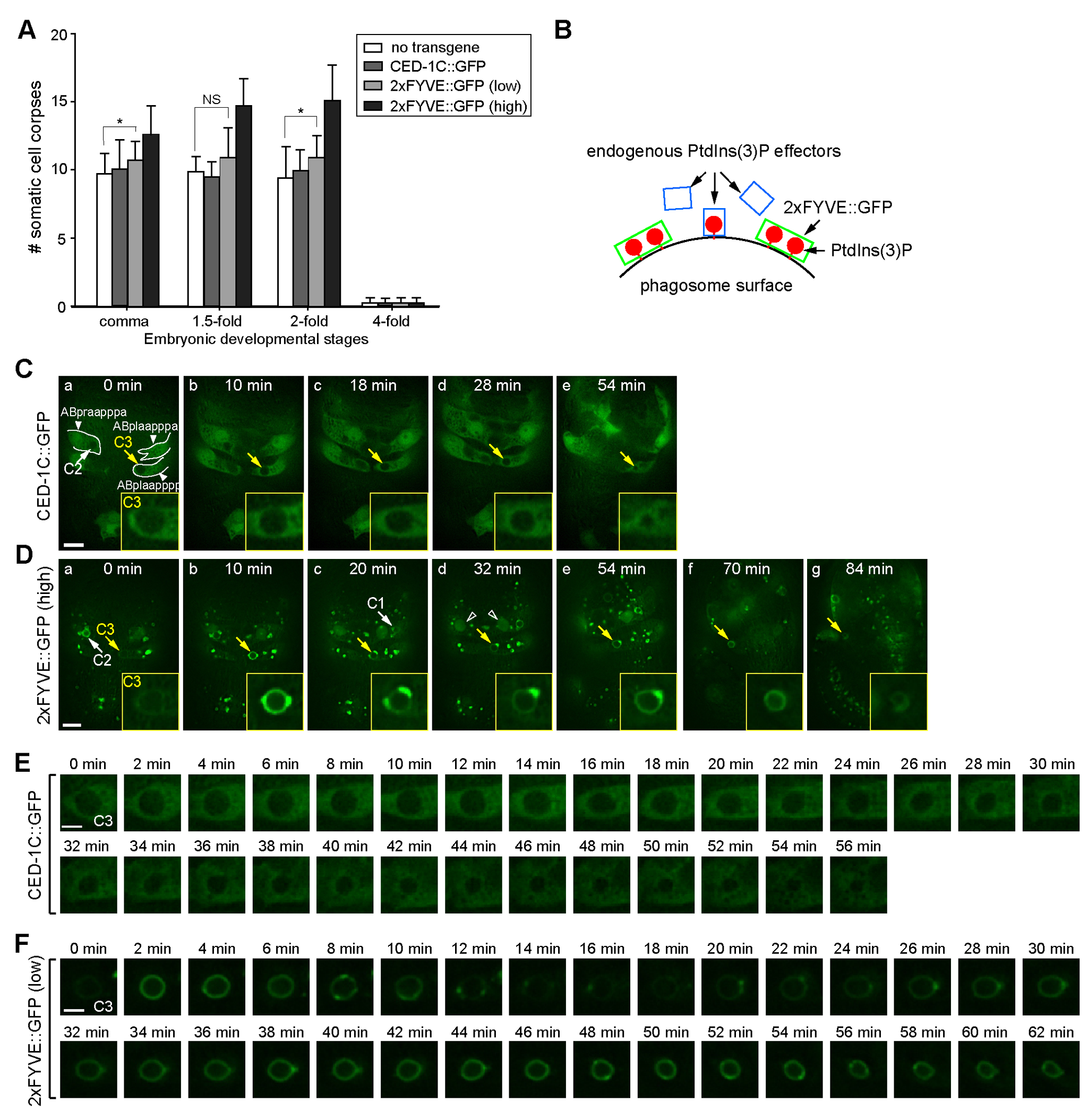

Supplement: Figure S6 — The sequestration effect of 2xFYVE::GFP reporter on phagosomal PtdIns(3)P is dependent on the expression level of the transgene (related to Figure 5). (A) The numbers of somatic cell corpses scored at different embryonic stages in wild-type embryos carrying indicated transgenes. High and low level of 2xFYVE::GFP expression was achieved by injecting worms with 20 ng/µl and 1 ng/µl plasmids, respectively. At least 20 embryos were scored for each data point. Data are presented as mean ± SD. *p<0.05, independent Student's t-test. “NS” indicates non-significant differences. (B) A diagram showing that the over-expressed 2xFYVE::GFP molecules may compete with endogenous PtdIns(3)P effectors for the interaction with phagosomal PtdIns(3)P. (C–D) Time-lapse images of the degradation of C3 phagosomes in wild-type embryos expressing transgenes Pced-1 CED-1C::gfp(C) or Pced-1 2xFYVE::gfp at a relatively high level. (D) “0 min” represents the time point when a C3 cell corpse was just fully internalized by its engulfing cell, ABplaapppp, and the newly formed phagosome was recognizable as a dark sphere inside GFP(+) engulfing cell. Anterior is to the top. Ventral faces readers. Arrows indicate cell corpses C1, C2, or C3; arrowheads indicate their corresponding engulfing cells; open arrowheads in D(d) indicate nuclei. Scale bars, 5 µm. (E–F) Time-lapse images of the degradation of C3 phagosomes in wild-type embryos expressing transgenes Pced-1 CED-1C::gfp (E) or Pced-1 2xFYVE::gfp at low level (F). “0 min” is when engulfment just completed. Scale bars, 2 µm. (TIF) [file pbio.1001245.s006.tif]

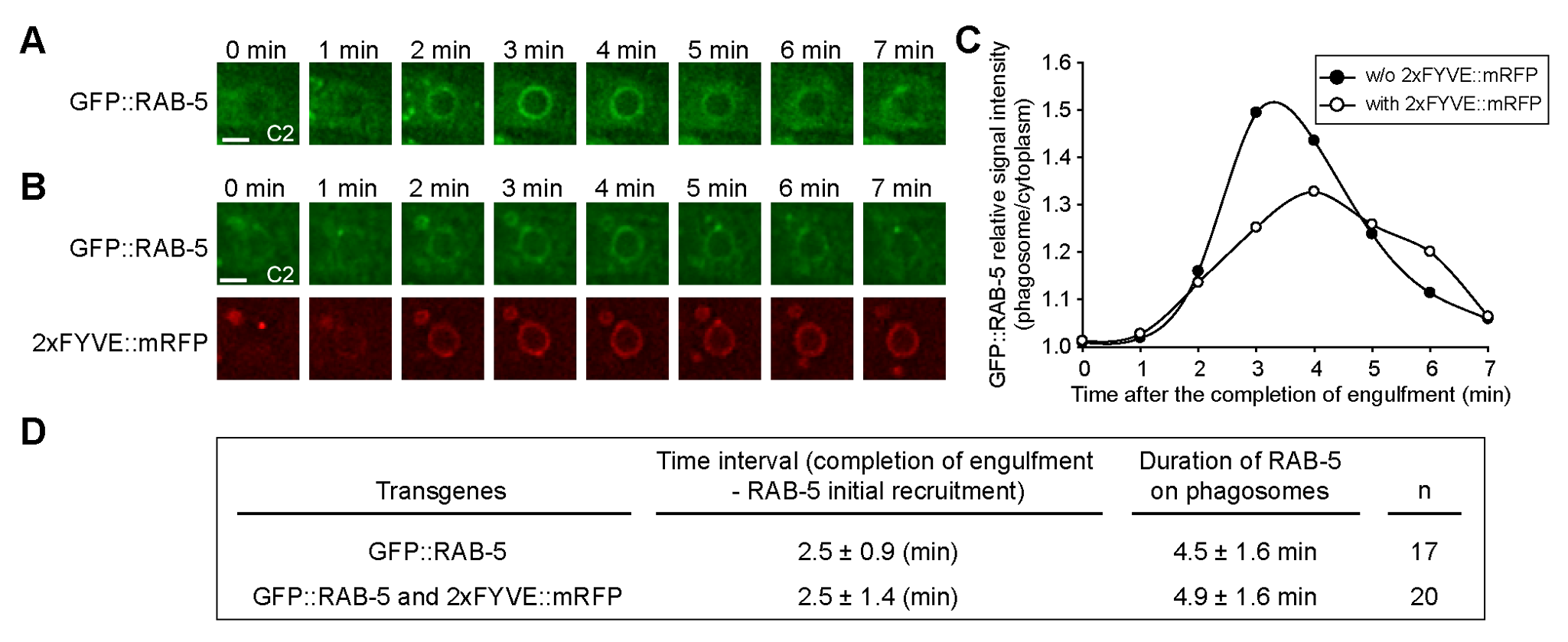

Supplement: Figure S7 — The timing of the transient enrichment of RAB-5 on nascent phagosomes is not affected by the expression of the 2xFYVE::GFP reporter. (A–B) Time-lapse recording of the dynamic phagosomal localization of GFP::RAB-5 on C2 phagosomes in wild-type embryos that expressed GFP::RAB-5 alone (A) or that co-expressed GFP::RAB-5 and high level of 2xFYVE::mRFP (B). “0 min” represents the time point when engulfment is just complete. Scale bars, 2 µm. (C) The relative GFP::RAB-5 signal intensity, represented as the ratio of GFP::RAB-5 signal intensity on the surface of phagosomes to that in the nearby cytoplasm of the host cell, was measured from images in (A–B) and plotted over time. (D) Quantification of the timing of the initial appearance of RAB-5 on phagosomes and the duration of RAB-5 in association with phagosomes. Data are presented as mean ± SD. n, the number of C1, C2, and C3 phagosomes analyzed. (TIF) [file pbio.1001245.s007.tif]

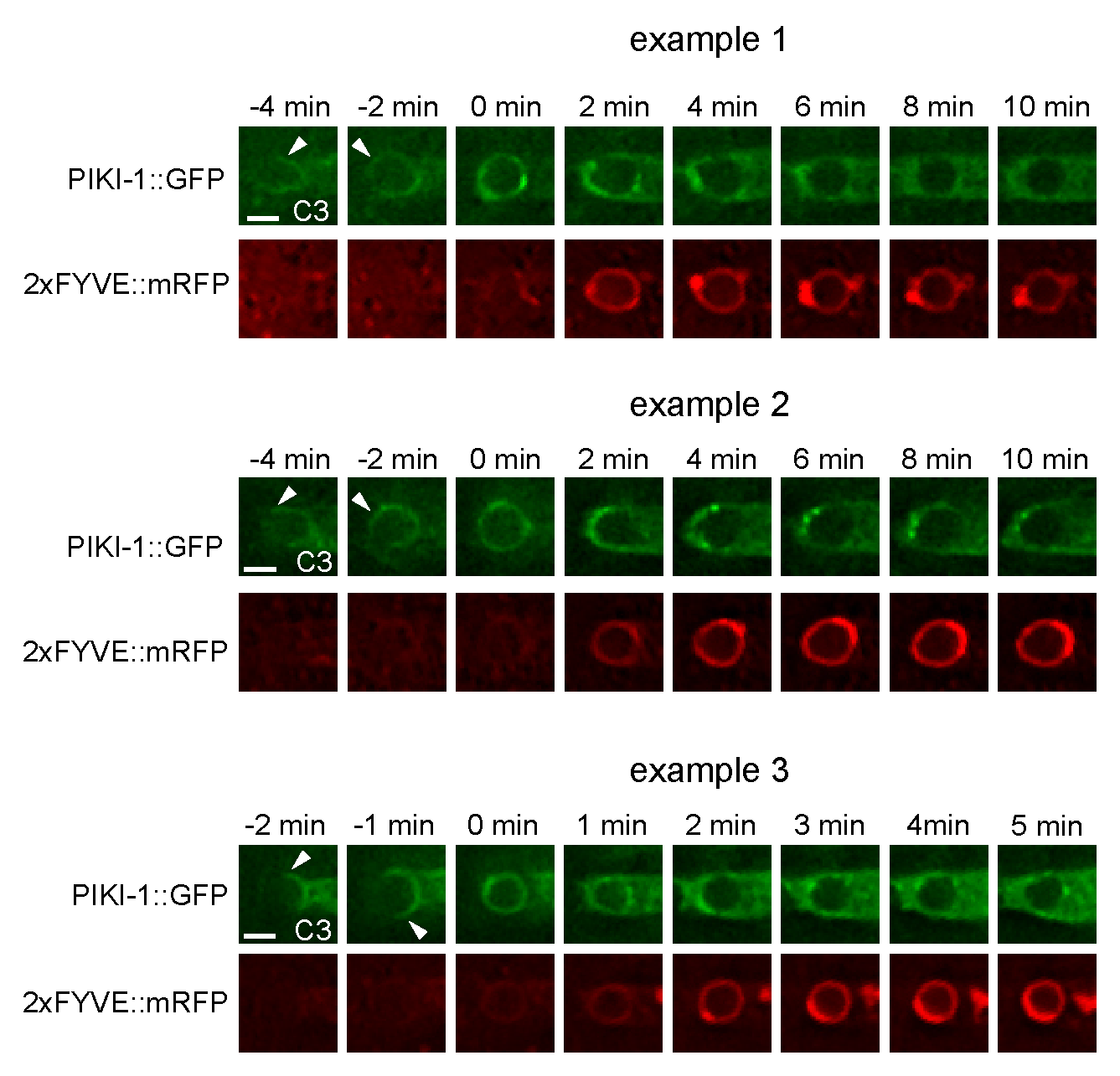

Supplement: Figure S8 — Three additional examples of the recruitment of PIKI-1 to the extending pseudopods and nascent phagosomes prior to the production of phagosomal PtdIns(3)P (related to Figure 6). Three time-lapse image series of the C3 phagosome in wild-type embryos co-expressing PIKI-1::GFP and 2xFYVE::mRFP. “0 min” represents the time point when engulfment is just complete. Arrowheads indicate the extending pseudopods. Scale bars, 2 µm. (TIF) [file pbio.1001245.s008.tif]

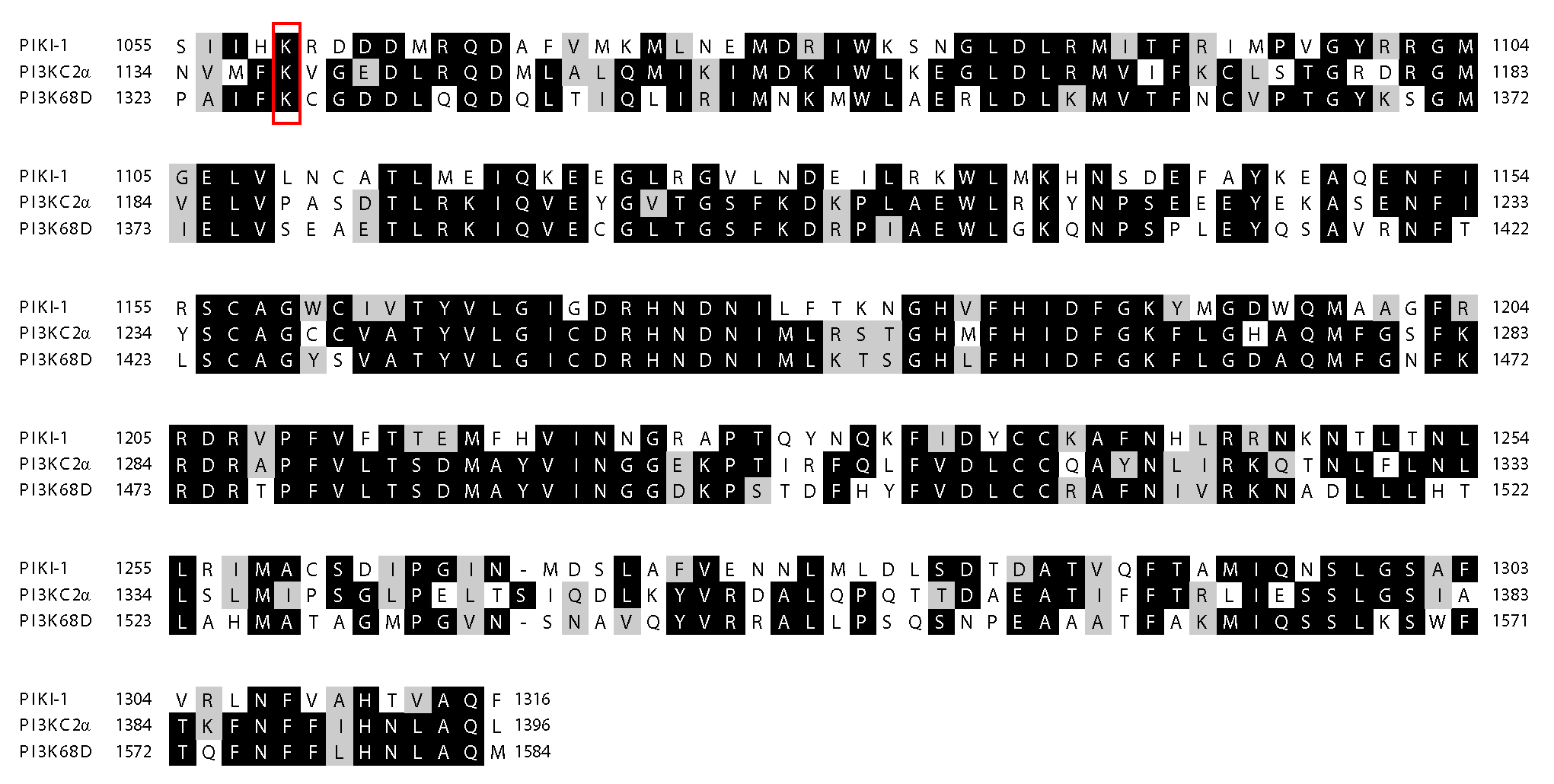

Supplement: Figure S9 — Sequence alignment of the kinase domain of class II PI 3-kinases that include C. elegans PIKI-1, H. sapiens PI3KC2α, and D. melanogaster PI3K68D (related to Figure 6). Lysine 1059, the conserved residue in the ATP binding motif, which was mutated in Pced-1piki-1(K1059A)::gfp, is labeled by a red frame. (TIF) [file pbio.1001245.s009.tif]

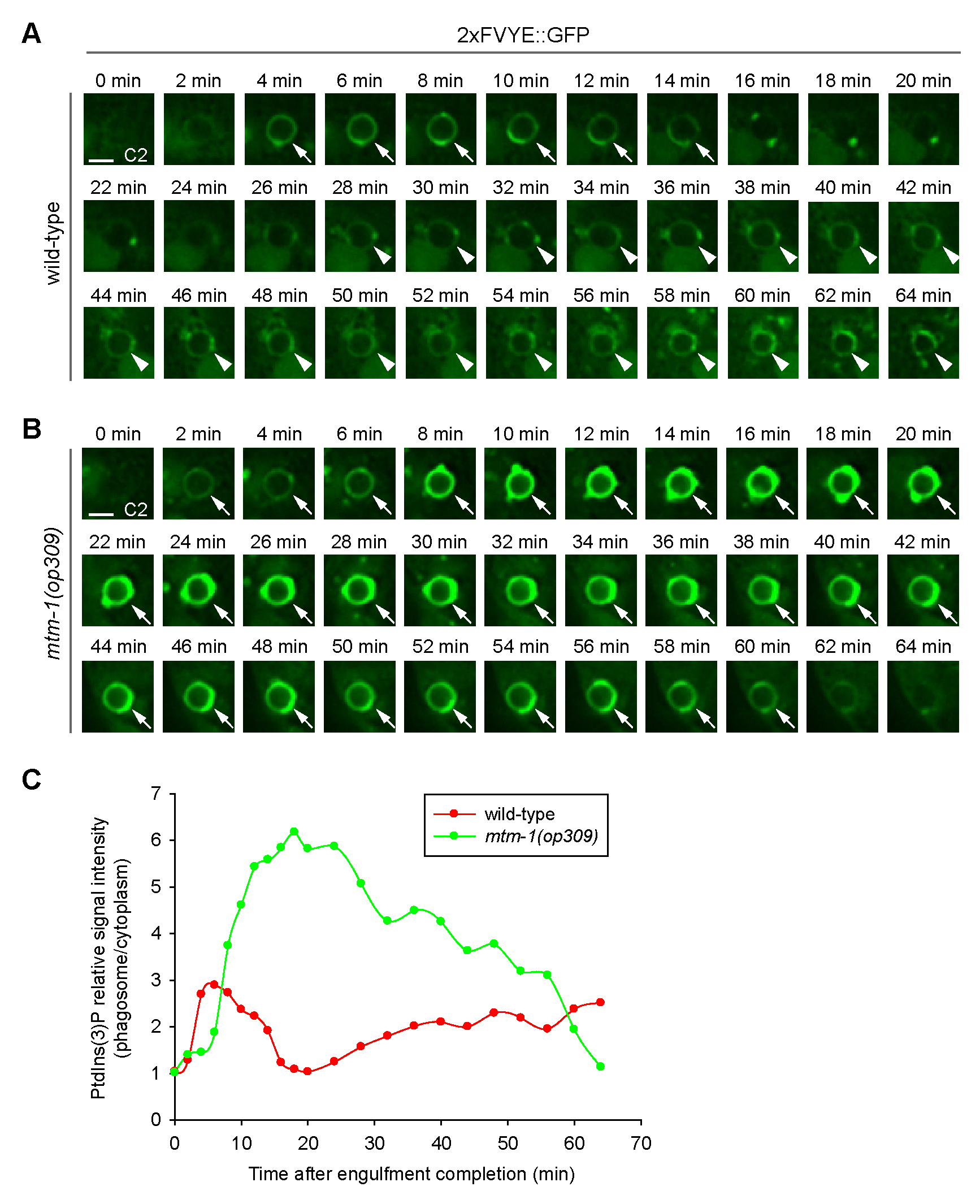

Supplement: Figure S10 — Additional examples of the enhanced and prolonged PtdIns(3)P signal on the phagosomes in mtm-1(op309) mutants (related to Figure 7). (A–B) The temporal presentation patterns of PtdIns(3)P on C2 phagosomes in a wild-type (A) or a mtm-1(op309) mutant (B) embryo were monitored by 2xFYVE::GFP. “0 min” is the time point when engulfment is just completed. Arrows and arrowheads indicate the phagosome maturation stages covered by the first and the second waves of PtdIns(3)P on phagosomes, respectively. Scale bars, 2 µm. (C) The relative PtdIns(3)P signal intensity, represented as the ratio of 2xFYVE::GFP signal intensity on the surface of phagosomes to that in the nearby cytoplasm of the host cell, was measured from images in (A–B) and plotted over time. (TIF) [file pbio.1001245.s010.tif]
