# Supplementary material for: Two PI 3-Kinases and One PI 3-Phosphatase Together Establish the Cyclic Waves of Phagosomal PtdIns(3)P Critical for the Degradation of Apoptotic Cells
Source: PLoS Biol. 2012 Jan 17;10(1):e1001245. doi: 10.1371/journal.pbio.1001245 (PMC3260314; doi:10.1371/journal.pbio.1001245)
Supplement: Text S1 — Supplemental text includes the following sections. Depletion of maternal and zygotic vps-34 gene products; characterization of piki-1 deletion alleles; normal endocytosis in piki-1 mutants; the requirement of RAB-5 for phagosomal PtdIns(3)P production; and the characterization of the effect of SNX-1::GFP, LST-4::GFP and 2xFYVE::GFP overexpression on phagosome maturation. (DOCX) [file pbio.1001245.s011.docx]

**Supplemental Material**

**Two PI3 Kinases and One PI3 Phosphatase Together Establish the Cyclic Waves of Phagosomal PtdIns(3)P Critical for the Degradation of Apoptotic Cells**

Nan Lu^1^, Qian Shen^1^, Timothy R. Mahoney^2^, Lukas J. Neukomm^3,4^, Ying Wang^1^, and Zheng Zhou^1,5,6^

**Supplemental Text**

**Depletion of both the maternal and zygotic *vps-34* products resulted in a complete loss of *vps-34* function**

*vps-34(h510)* is a null mutation [1]. *vps-34(h510)* homozygous progeny of *vps-34(h510)/+* heterozygous mothers, which carry the maternal *vps-34* gene product, undergo larval development arrest [1]*.* To further deplete the maternal *vps-34* product and create homozygous *vps-34(h510)(m^-^z^-^)* (*m* and *z*: maternal and zygotic gene products, respectively) embryos, we constructed a strain in which the homozygous *vps-34(h510)* mutants were kept alive and fertile by an extrachromosomal array carrying the *vps-34(+)* genomic DNA and an mRFP marker expressed in embryos. Extrachromosomal arrays are lost at a certain frequency during meiosis [2]. In addition, transgene expression is usually repressed in the hermaphrodite germline due to germline silencing of repetitive extrachromosomal arrays [3,4]. Therefore, the mRFP(-) (*vps-34(h510)(m^-^z^-^))* progeny of the *vps-34(h510)*; *enEx[vps-34(+), mRFP marker]* mothers, from which the extrachromosomal array is lost, are unlikely to inherit much maternal *vps-34(+)* product and at the same time lack zygotic product of *vps-34*.

*vps-34(h510)(m^-^z^-^)* homozygous mutant animals displayed a much severer developmental defect than *vps-34(h510)(m^+^z^-^)* homozygous mutant animals descended from *vps-34(h510)/+* heterozygous mothers. In particular, whereas *vps-34(h510)(m^+^z^-^)* animals underwent developmental arrest primarily at the L3 larval stage [1], ~75% of *vps-34(h510)(m^-^z^-^)* animals underwent an earlier developmental arrest during embryogenesis (Figure S1B). This phenomenon indicates that as expected, the *vps-34(h510)(m^-^z^-^)* homozygous mutant phenotypes truly reflect the physiological consequences resulted from the complete loss of *vps-34* gene function. We thus used *vps-34(h510)(m^-^z^-^)* embryos for all further analyses regarding the function of *vps-34* in embryos.

***piki-1* deletions cause strong Ced phenotypes**

The gene structure of *piki-1* is presented in Figure S2A. To determine the loss-of-function phenotypes of *piki-1*, we characterized two deletion alleles, *piki-1(tm3171)* (obtained from http://www.shigen.nig.ac.jp/c.elegans/index.jsp) and *piki-1(ok2346)* (obtained from http://www.cbs.umn.edu/CGC/) (Figure S2A). *tm3171* is a deletion allele that removes 673bp DNA sequence that encodes a large portion of the catalytic domain of PIKI-1 (Figure S2B) and is likely to represent a null allele of *piki-1*. The *ok2346* allele carries a 1597 bp-deletion of the *piki-1* genomic DNA, a deletion that removes the PIK (PI-kinase signature) domain and part of the catalytic domain, and is thus predicted to encode a truncated PIKI-1 protein missing the entire PIK domain and a functional catalytic domain (Figure S2B).

Both *piki-1(tm3171)* and *piki-1(ok2346)* homozygous mutants are viable and fertile, with no detectable developmental defects. To examine the function of PIKI-1 in cell-corpse removal, we scored the numbers of cell corpses in *piki-1(tm3171)* and *piki-1(ok2346)* embryos and adult hermaphrodite gonads. We found that embryos bearing either mutation contained significantly larger numbers of cell corpses than wild-type embryos, starting from mid-embryogenesis, indicating a defect in the removal of cell corpses (Ced phenotype) (Figure S2E). A much stronger Ced phenotype was also observed in the gonad of adult hermaphrodites in both *piki-1(tm3171)* and *piki-1(ok2346)* single mutant backgrounds (Figure S2 C and D). The phenotypes displayed by *piki-1(tm3171)* and *piki-1(ok2346)* single mutant animals are comparable in severity (Figure S2 D and E). Together, the strong Ced phenotypes and the length and position of the deleted fragments strongly suggest that both the *tm3171* and *ok2346* alleles are likely to be null alleles of *piki-1*.

***piki-1* mutants are normal in endocytosis**

Unlike *vps-34(h510)* mutants [1], *piki-1(tm3171)* deletion mutants are both viable and fertile. To determine whether PIKI-1 specifically promotes the degradation of apoptotic cells or, like VPS-34, also acts in other cellular events, we examined whether endocytosis, a process that requires the function of PtdIns(3)P [reviewed in 5], is defective in *piki-1(tm3171)* mutants. We first examined the uptake of yolk by *piki-1* mutant embryos and the redistribution of yolk in embryonic tissues during embryogenesis. In *C. elegans*, yolk, a large lipoprotein complex produced by the intestinal cells of the mother, is transported to the mother’s gonad, where it is internalized by oocytes through a clathrin-mediated pathway [6]. During embryogenesis, yolk is secreted from most embryonic cell types through exocytosis and internalized by intestinal precursor cells [7]. We examined whether, in *piki-1* mutants, yolk was internalized by oocytes and underwent subsequent redistribution during embryogenesis by following YP170::GFP [6], a yolk reporter produced by adult hermaphrodites. Consistent with previous reports [6,7], in wild-type animals, maternally produced YP170::GFP was internalized by oocytes (Figure S3A). During early embryonic stages (<200 cells), yolk was observed evenly distributed in cells (Figure S3B(a, d)). Later, yolk gradually accumulated in intestinal precursor cells and meanwhile it diminished from most other cell types (Figure S3B(b, e), demonstrating the occurrence of yolk redistribution process. By the 2-fold stage, yolk disappeared from the entire head region and was highly enriched in the intestinal precursor cells (Figure S3B(c, f)). In *piki-1(tm3171)* mutants, we observed the normal disposition of YP170::GFP into oocytes and the normal redistribution of YP170::GFP during embryogenesis (Figure S3 A-B). Our observations indicate that yolk endocytosis and exocytosis are both normal in *piki-1(tm3171)* mutants.

We next examined the endocytosis activity of coelomocytes, six scavenger cells that are localized in the pseudocoelom (body cavity) of each worm and nonspecifically endocytose body waste and fluid [8]. A secreted GFP reporter (ssGFP), expressed under the control of a body wall muscle-specific promoter (P*_myo-3_*) and secreted into the body cavity, is observed at a very low level inside the body but at high levels inside coelomocytes in wild-type animals, indicating that coelomocytes actively endocytosed ssGFP from the body cavity [8]. Using the P*_myo-_*_3_ssGFP reporter, it was found that RNAi inactivation of *vps-34* resulted in the block of coelomocyte endocytosis of ssGFP, indicating that VPS-34 is essential for the endocytosis activity of VPS-34 [8]. By monitoring the localization of the P*_myo-_*_3_ssGFP reporter, we observed the same distribution pattern of ssGFP in both wild-type and *piki-1(tm3171)* mutant animals: ssGFP was largely accumulated in the coelomocytes and almost undetectable in other parts of the body (Figure S3 C-D). These observations indicate that coelomocytes possess normal endocytosis activity in *piki-1(tm3171)* mutant background.

Together, the above results demonstrate that unlike VPS-34, PIKI-1 is not involved in endocytosis events, whether it is receptor-mediated yolk uptake by oocytes and yolk redistribution in embryos or non-specific fluid endocytosis by coelomocytes.

**RAB-5 positively regulates the production of PtdIns(3)P on phagosomal surfaces**

To further dissect the relationships between PtdIns(3)P and the RAB GTPases that are involved in phagosome maturation, we examined the presentation of PtdIns(3)P on phagosomes in each of the *rab-5*, *rab-2* and *rab-7* loss-of-function background. The inactivation of *rab-5*, *rab-2* or *rab-7* by *rab-5*(RNAi), a strong loss-of-function mutation *rab-2(n3263),* or a null mutation *rab-7(ok511)*, respectively, significantly increased the numbers of germ cell corpses in adult hermaphrodites (Figure S4A), verifying the essential function of these RAB GTPases in the degradation of cell corpses as previously reported [9,10,11,12,13]. In the gonads of *rab-2(n3263)* or *rab-7(ok511)* mutant adult hermaphrodites, PtdIns(3)P was presented on the surfaces of phagosomes at normal frequencies and levels (Figures 4E and S4), indicating *rab-2* and *rab-7* are not required for PtdIns(3)P production on phagosomes. In contrast, in the gonads of *rab-5(RNAi)* treated adult hermaphrodites, only 32 % of phagosomes were labeled by PtdIns(3)P, reduced from 86% in the wild-type background (Figures 4E and S4(b, f)), indicating that the production of PtdIns(3)P on phagosome depends on RAB-5 function. The mutual dependence of PtdIns(3)P and RAB-5 on each other’s function for phagosomal enrichment is consistent with the concurrent localization of PtdIns(3)P and RAB-5 on nascent phagosomes (Figure S7) [14].

**Do the SNX-1::GFP and LST-4::GFP reporters cause defects in phagosome maturation?**

To determine whether the GFP reporters for the dynamic phagosomal localization of SNX-1 and LST-4 (Figure 3), which are overexpressed in engulfing cells as extrachromosomal arrays, affect the progress of phagosome maturation, we characterized two aspects related to phagosome maturation in wild-type embryos expressing each of SNX-1::GFP and LST-4::GFP. First we scored the number of cell corpses in each strain, including a no-transgene control, at 4-different embryonic stages (Figure S5A). We found that the expression of SNX-1::GFP did not increase the number of cell corpses comparing to the no-transgene control, indicating no dominant-negative effect in the removal of cell corpses (Figure S5A). The LST-4::GFP reporter caused very modest increases (15% and 26%) in the number of cell corpses in two of the four stages that are scored (Figure S5A). We next measured the duration of embryonic phagosomes using time-lapse recording and noticed ~5 min increases in the mean duration of phagosomes caused by each of the reporters (Figure S5B). Both reporters appeared to slightly increase the variability of the length of phagosome duration (Figure S5B). Overall, neither reporter elicits a substantial effect over phagosome maturation.

**The effects of the 2xFYVE::GFP reporter in engulfing cells**

**1. The sequestration of phagosomal PtdIns(3)P slows down the degradation of apoptotic cells in a dosage-dependent manner**

In *C. elegans*, a transgene is maintained in the form of an extrachromosomal array, which contains multiple tandem copies of the reporter construct [2]. As a result, transgenes are usually overexpressed in somatic tissues. In wild-type animals carrying an extrochromosomal array expressing P*_ced-1_* *2xFVYE::gfp*, we observed a larger number of germ cell corpses in the gonad of adult hermaphrodites in comparison to wild-type animals not carrying any transgene (Figure S1A). Furthermore, in wild-type embryos carrying P*_ced-1_* *2xFVYE::gfp* at a relatively high concentration (microinjected at 20ng/μl into the mother’s gonad), significantly larger number of cell corpses were observed at three mid-embryogenesis stages, although not at the late 4-fold stage, the last stage before hatching, in comparison to wild-type embryos not carrying any transgene, or carrying P*_ced-1_* *ced-1C::gfp*, which encoded a cytoplasmic GFP reporter (Figure S6A). In wild-type embryos carrying P*_ced-1_* *2xFVYE::gfp* at a low concentration (microinjected at 1ng/μl into the mother’s gonad), much weaker effects were observed (Figure S6A). These observations suggest that the 2xFYVE::GFP protein, which is presumably overexpressed in engulfing cells and is observed to associate with phagosomal PtdIns(3)P, perturbs the removal of cell corpses in a dosage-dependent manner. These results further suggest that 2xFYVE::GFP might elicit this effect through sequestering phagosomal PtdIns(3)P from its endogenous effectors that promote phagosome maturation (Figure S6B).

To test the above hypothesis, we measured the duration of phagosomes, the time interval from when a phagosome first formed to when it shrunk to an undetectable size, using a previously establish time-lapse recording approach [15,16]. We chose to measure the duration of phagosomes that contain cell corpses C1, C2 and C3, which were located on the ventral surface, died almost simultaneously during mid-embryogenesis, and were quickly engulfed and degraded by three adjacent hypodermal cells that are extending to the ventral midline (Figure S6C(a)) [15,16]. The duration of phagosomes was prolonged to different extents, depending on the overexpression level of 2xFYVE::GFP , in comparison to the duration of the same phagosomes in wild-type embryos expressing P*_ced-1_* *ced-1C*::gfp, a cytosolic GFP reporter that allowed the recognition of phagosomes as GFP(-) dark spheres inside green engulfing cells yet did not interfere with phagosome maturation (Figure S6 A and C-F) [17]. Our observations thus indicate that sequestering phagosomal PtdIns(3)P slows down phagosome maturation in a dosage-dependent manner.

**2. The oscillation pattern of phagosomal PtdIns(3)P is not affected by the overexpression of the 2xFYVE::GFP reporter**

Previously, using a 2xFYVE::GFP (injected at 20ng/μl)) reporter, we observed a temporal oscillation pattern of PtdIns(3)P throughout the phagosome maturation process (Figure 5A) [9,11]. In order to determine whether this oscillation pattern reflected the real PtdIns(3)P localization pattern on phagosomes or was a result of the dominant-negative effect of 2xFYVE::GFP, we generated multiple transgenic lines that carried the P*_ced-1_* *2xFYVE::gfp* reporter at a much lower concentration (injected at 1ng/μl). Our time-lapse recording results obtained from monitoring multiple phagosomes indicate that when expressed at a presumably much lower level, 2xFYVE::GFP was still able to detect the same oscillation pattern of phagosomal PtdIns(3)P (Figure S6F and data not shown). Virtually, the timing of the initial and subsequent appearance of PtdIns(3)P on phagosomes and the durations of the first PtdIns(3)P wave and the PtdIns(3)P(-) gap are comparable to that obtained using the 2xFYVE::GFP reporter injected at higher concentration. These results indicate that the levels of 2xFYVE::GFP present in the host cells do not affect the oscillation pattern of PtdIns(3)P.

**3. The timing of the transient enrichment of RAB-5 on nascent phagosomes is not affected by the 2xFYVE::GFP reporter**

The severe reduction of PtdIns(3)P production activity, as a result of the *piki-1(tm3171)* null mutation, impaired the recruitment of GFP::RAB-5 to phagosomal surface (see text, Figure 4). To examine whether sequestrating phagosomal PtdIns(3)P by the overexpressed 2xFYVE::mRFP would affect the recruitment of RAB-5, the GFP::RAB-5 reporter was co-expressed with a relatively high concentration of 2xFYVE::mRFP (injected at 20ng/μl) in engulfing cells, and the kinetics of RAB-5’s association with multiple phagosomes was monitored by time-lapse recording. We found that, in comparison to the condition in which GFP::RAB-5 was expressed alone, the co-expression of 2xFYVE::mRFP did not significantly change the kinetics of RAB-5 recruitment (Figure S7). In particular, the timing of the initial appearance of RAB-5 on phagosomes and the duration of RAB-5 in association with phagosomes are similar in the absence or presence of the 2xFYVE::mRFP reporter (Figure S7 A, B and D). On the other hand, quantitative measurement of the GFP::RAB-5 signal intensities indicates that the presence of 2xFYVE::mRFP reduced the peak level of RAB-5 on phagosomes (Figure S7 A-C). This reduction is consistent with the proposed role of 2xFYVE::mRFP in sequestrating phagosomal PtdIns(3)P from other phagosome maturation effectors, and is likely to be one of the causes for the modest Ced phenotype observed from wild-type animals expressing a high level of 2xFYVE reporters (Figure S6A).

**References**

1. Roggo L, Bernard V, Kovacs AL, Rose AM, Savoy F, et al. (2002) Membrane transport in Caenorhabditis elegans: an essential role for VPS34 at the nuclear membrane. Embo J 21: 1673-1683.

2. Jin Y (1999) Transformation. In: Hope IA, editor. C elegans, a practical approach.

Oxford: Oxford University Press. pp. 69-96.

3. Kelly WG, Fire A (1998) Chromatin silencing and the maintenance of a functional

germline in Caenorhabditis elegans. Development 125: 2451-2456.

4. Kelly WG, Xu S, Montgomery MK, Fire A (1997) Distinct requirements for somatic

and germline expression of a generally expressed Caernorhabditis elegans gene. Genetics 146: 227-238.

5. Backer JM (2008) The regulation and function of Class III PI3Ks: novel roles for Vps34. Biochem J 410: 1-17.

6. Grant B, Hirsh D (1999) Receptor-mediated endocytosis in the Caenorhabditis elegans oocyte. Mol Biol Cell 10: 4311-4326.

7. Bossinger O, Schierenberg E (1996) The use of fluorescent marker dyes for studying intercellular communication in nematode embryos. Int J Dev Biol 40: 431-439.

8. Fares H, Greenwald I (2001) Genetic analysis of endocytosis in Caenorhabditis elegans: coelomocyte uptake defective mutants. Genetics 159: 133-145.

9. Yu X, Lu N, Zhou Z (2008) Phagocytic receptor CED-1 initiates a signaling pathway for degrading engulfed apoptotic cells. PLoS Biol 6: e61.

10. Kinchen JM, Doukoumetzidis K, Almendinger J, Stergiou L, Tosello-Trampont A, et al. (2008) A pathway for phagosome maturation during engulfment of apoptotic cells. Nat Cell Biol 10: 556-566.

11. Mangahas PM, Yu X, Miller KG, Zhou Z (2008) The small GTPase Rab2 functions in the removal of apoptotic cells in Caenorhabditis elegans. J Cell Biol 180: 357-373.

12. Lu Q, Zhang Y, Hu T, Guo P, Li W, et al. (2008) C. elegans Rab GTPase 2 is required for the degradation of apoptotic cells. Development 135: 1069-1080.

13. Li W, Zou W, Zhao D, Yan J, Zhu Z, et al. (2009) C. elegans Rab GTPase activating protein TBC-2 promotes cell corpse degradation by regulating the small GTPase RAB-5. Development 136: 2445-2455.

14. Zhou Z, Yu X (2008) Phagosome maturation during the removal of apoptotic cells: receptors lead the way. Trends Cell Biol 18: 474-485.

15. Yu X, Odera S, Chuang CH, Lu N, Zhou Z (2006) C. elegans Dynamin mediates the signaling of phagocytic receptor CED-1 for the engulfment and degradation of apoptotic cells. Dev Cell 10: 743-757.

16. Lu N, Yu X, He X, Zhou Z (2009) Detecting apoptotic cells and monitoring their clearance in the nematode Caenorhabditis elegans. Methods Mol Biol 559: 357-370.

17. He B, Yu X, Margolis M, Liu X, Leng X, et al. (2010) Live-Cell Imaging in Caenorhabditis elegans Reveals the Distinct Roles of Dynamin Self-Assembly and Guanosine Triphosphate Hydrolysis in the Removal of Apoptotic Cells. Mol Biol Cell 21: 610-629.
